# Robust simplifications of multiscale biochemical networks

**DOI:** 10.1186/1752-0509-2-86

**Published:** 2008-10-14

**Authors:** Ovidiu Radulescu, Alexander N Gorban, Andrei Zinovyev, Alain Lilienbaum

**Affiliations:** 1 IRMAR (CNRS UMR 6025) and IRISA/INRIA, Université de Rennes 1, Campus de Beaulieu, 35042 Rennes, France; 2 Department of Mathematics, University of Leicester, LE1 7RH, UK; 3 Institut Curie, Service Bioinformatique, Paris, 26 rue d'Ulm, Paris F-75248, France; 4 INSERM, U900, Paris, F-75248, France; 5 Ecole des Mines de Paris, ParisTech, Fontainebleau, F-77300, France; 6 Institute of Computational Modeling SB RAS, Krasnoyarsk, Akademgorodok, 660036, Russia; 7 Cytosquelette et Développement (CNRS UMR 7000), Faculté de Médecine Pitié-Salpêtrière, 105, boulevard de l'Hôpital, 75634 Paris cedex 13, France; 8 Stress et Pathologies du Cytosquelette (EA300), Université Paris 7 Denis Diderot, 4, rue Marie-Andrée Lagroua Weill-Hallé 75013 Paris, France

## Abstract

**Background:**

Cellular processes such as metabolism, decision making in development and differentiation, signalling, etc., can be modeled as large networks of biochemical reactions. In order to understand the functioning of these systems, there is a strong need for general model reduction techniques allowing to simplify models without loosing their main properties. In systems biology we also need to compare models or to couple them as parts of larger models. In these situations reduction to a common level of complexity is needed.

**Results:**

We propose a systematic treatment of model reduction of multiscale biochemical networks. First, we consider linear kinetic models, which appear as "pseudo-monomolecular" subsystems of multiscale nonlinear reaction networks. For such linear models, we propose a reduction algorithm which is based on a generalized theory of the limiting step that we have developed in [1]. Second, for non-linear systems we develop an algorithm based on dominant solutions of quasi-stationarity equations. For oscillating systems, quasi-stationarity and averaging are combined to eliminate time scales much faster and much slower than the period of the oscillations. In all cases, we obtain robust simplifications and also identify the critical parameters of the model. The methods are demonstrated for simple examples and for a more complex model of NF-*κ*B pathway.

**Conclusion:**

Our approach allows critical parameter identification and produces hierarchies of models. Hierarchical modeling is important in "middle-out" approaches when there is need to zoom in and out several levels of complexity. Critical parameter identification is an important issue in systems biology with potential applications to biological control and therapeutics. Our approach also deals naturally with the presence of multiple time scales, which is a general property of systems biology models.

## Background

Model reduction techniques are used to reduce the dimensionality of complex dynamics. Applications of model reduction techniques in chemical engineering (coarse graining in phase transitions, reactors, combustion [2-8]), in ecology [9] or climatology, are well developed. A collection of reviews in model reduction for kinetic problems can be found in [10]. In systems biology, ad hoc reduction methods have been applied to signal transduction [11] and to clocks [12,13]. Combinatorial complexity of receptors and scaffolds can be reduced by exact lumping [14,15].

We may distinguish among three classes of model reduction techniques. *Trajectory based techniques *use the integration of the dynamical equations and look for a small number of reduced variables [16]. The empirical orthogonal eigenfunctions (EOF), also called Proper Orthogonal Decomposition (POD), or Karhunen-Loève expansion (KL) method, consists in finding a low dimension linear (flat) manifold, containing (or sufficiently close to) the trajectories [17,18]. *Singular perturbations techniques *eliminate fast variables whose dynamics is slaved by the slower variables. The Computational Singular Perturbation (CSP) method provides approximations of a low dimensional invariant manifold, containing the dynamics [2,3]. Invariant manifolds can be calculated by various other methods [4-8]. Slow-fast or more general master-slave splittings (splittings with no feed-back) were discussed by [19,20]. Chemical enzymatic kinetics beyond quasi-stationarity and quasi-equilibrium has been studied in [21]. Averaging has been used to eliminate rapid oscillations of microscopic degrees of freedom and to obtain smaller models [22-24]. *Aggregation or lumping techniques *have been proposed by many authors [9,14,15,25]. Reaction graph contraction methods such as Clarke's [26] replace the reactions mechanism by simpler mechanisms in which some intermediate species are absent.

Normally, identification of two well separated time scales is enough to reduce the system by using slow/fast decompositions [20]. However, the biochemical networks used to model cell physiology are multiscale, i.e. they have many, well separated time scales. For example, changing gene expression programs can take hours and even days while protein complex formation goes on the second scale and post-translational protein modifications take minutes to happen. Protein life half-times can vary from minutes to days. This important observation applies not only to time scales but also to concentration values of various species in these networks. mRNA copy numbers can change from some units to tens of thousands, and the dynamic concentration range of biological proteins can reach up to five orders of magnitude.

The aim of our paper is to propose model reduction methods well adapted to this situation. The mathematical techniques that we use (limitation, averaging, quasi-stationarity) have a long history. However, their combination into practical recipes that we propose is original and well adapted for the study of multiscale biochemical networks. Our most important development is the concept of dominant subsystem (that we also call limit simplification).

The idea of dominant subsystems in asymptotic analysis of dynamical systems is due to Newton and developed by Kruskal [27]. There are several ways to obtain dominant subsystems. These can be leading terms in power expansions of small parameters. Thus, multiscale expansions are standard techniques in perturbation theory [28]. Asymptotic theories using powers of small parameters were applied to study spectral properties of multiscale matrices [27,29-31]. In [1] we have proposed a different approach to dominant subsystems. This approach exploits the reaction network structure to select dominant pathways and to obtain simplified reaction mechanisms. The simplifications are robust because are valid for a large range of parameters.

Understanding the functioning of large networks of biochemical reactions could rely on having a hierarchy of such simplifications, ie a set of models that can be obtained one from another by model reduction. Molecular networks are designed to fulfill many simple tasks. For each one of this tasks, the system scans only a small part of its high dimensional phase space. Geometrically speaking, it evolves on a stable low dimensional invariant manifold with branching in the fast directions [5]. Changing tasks, the system can jump from one stable branch of the manifold to another one. These represent jumps from one simplification (dominant subsystem) to another one. Finding the set of simplifications of a molecular network means providing the set of functioning modes for the network.

Thus, dominant subsystems provide an answer to a very practical question: how to describe the dynamics of a multiscale network? During almost all time this could be simplified and the system behaves as a small one. Our methods show how to obtain the small dominant subsystem from the topology of the network and from the orders of magnitude of kinetic constants and species concentrations. In multiscale systems, concentration orders can change dynamically and the small system may change at discrete times. The whole system walks along small subsystems. The discrete dynamics of this walk supplements the dynamics of individual small subsystems.

Dominant subsystem can be used to answer another important question: given a network model, which are its critical parameters? Many of the parameters of the initial model are no longer present in the dominant subsystem: these parameters are non-critical. Parameters of dominant subsystems indicate putative targets to change the behavior of the large network.

Finally, dominant subsystems can be used to compare models. Systems biology model repositories contain models of various degree of complexity. To be compared, or to be integrated into larger ones, models must be simplified to a common level of complexity.

Our methods perform well when we have total or partial separation of time and/or concentration scales. Total separation of time scales means the following: picking two timescales at random *τ*_*i*_, *τ*_*j *_one has either *τ*_*i *_<<*τ*_*j *_or *τ*_*i *_>> *τ*_*j *_with probability close to one. It is easy to construct a totally separated linear system. Choose constants of biochemical reactions independently and distributed uniformly over a large interval in logarithmic scale: picking two timescales at random *τ*_*i*_, *τ*_*j *_one has either *τ*_*i *_<<*τ*_*j *_or *τ*_*i *_>> *τ*_*j *_with probability close to one. This situation has been studied in detail in [1]. Though, it is difficult to have total separation in non-linear systems. For these, even if reactions constants are independent, timescales are not. Our methods for robust simplifications of nonlinear systems functions also when scales are partially separated: in this case we gather terms of the same order in the quasi-stationarity and averaged steady state equations.

The models that we study here are deterministic. Reduction methods for stochastic multiscale biochemical kinetics can be found in [32,33].

The structure of this paper is the following. In the first section we present how to compute dominant subsystems for totally separated linear networks of (pseudo)monomolecular reactions. These appear as subsystems in analysis of multiscale networks of nonlinear biochemical reactions. This method uses the theory of limitation developed in [1]. In the second section, we show how to obtain dominant subsystems of non-linear systems. The technique is based on a method for identification of quasi-stationary and non-oscillating species and on dominant approximations of the quasi-stationarity and averaged steady-state equations for these species. In the third section, we introduce and analyze a new high dimensional model for the NF-*κ*B signalling.

## Methods

### Reduction of linear hierarchical models

#### Introductory notes

In this section we present a general algorithm for finding dominant subsystems and critical parameters for linear systems with completely separated time scales. Linear systems represent a special situation when all the interactions in the reaction network are monomolecular, i.e., have the form *A *→ *B*.

Although systems biology models are nonlinear and contain also multimolecular reactions, it is nevertheless useful to have an efficient algorithm for solving linear problems. First, as we shall see in the next section, non-linear systems can include linear subsystems, containing reactions that are pseudo(monomolecular) with respect to species internal to the subsystem (at most one internal species is reactant and at most one is product). Second, for reactions *A *+ *B *→ ..., if concentrations *c*_*A *_and *c*_*B *_are well separated, say *c*_*A *_>> *c*_*B*_, then we can consider this reaction as *B *→ ... with rate constant proportional to *c*_*A *_which is practically constant, or changes only slowly. We can assume that this condition is satisfied for all but a small fraction of genuinely non-linear reactions (the set of non-linear reactions changes in time but remains small). Thus, linear models can serve as very effective approximations of behavior of non-linear models in certain windows of time: in this way, non-linear behavior can be approximated as a sequence of linear dynamics, followed one each other in a sequence of "phase transitions". Third, linear networks represent the case when very large reaction networks models can be approached analytically, and some intuition and design principles can be learned and partially generalized to the non-linear case. As an example, see the robustness study made in [34]. The linear case offers nice simple illustrations of the concepts of dominant subsystem, critical monomials and critical parameters.

The algorithm presented here in its "recipe" form ready for computational implementations, is developed in detail elsewhere [34], with many examples and rigorous justifications.

The structure of linear (monomolecular) reaction networks can be completely defined by a simple digraph, in which vertices correspond to chemical species *A*_*i*_, edges correspond to reactions *A*_*i *_→ *A*_*j *_with kinetic constants *k*_*ji *_> 0. For each vertex, *A*_*i*_, a positive real variable *c*_*i *_(concentration) is defined.

"Pseudo-species" (labeled ∅) can be defined to collect all degraded products, and degradation reactions can be written as *A*_*i *_→ ∅ with constants *k*_0*i*_. Production reactions can be represented as ∅ → *A*_*i *_with rates *k*_*i*0_.

The kinetic equation is

(1) $$\frac{dc_{i}}{dt}=k_{i0}+\sum_{j\geq 1}k_{ij}c_{j}-(\sum_{j\geq 0}k_{ji})c_{i},$$

or in vector form: *ċ *= *K*_0 _+ *Kc*.

The advantage of linear dynamics is that it is completely specified by the eigenvectors and the eigenvalues of the kinetic matrix *K*.

The system has an unique bounded steady state *c*^*s *^= *K*^-1 ^*K*_0 _if and only if the matrix *K *is non-singular.

In this case, it is easy to write down the general solution of Eq.(1):

(2) $$c(t)=c^{s}+\sum_{k=1}^{n}r^{k}(l^{k},c(0)-c^{s})\exp(-\lambda_{k}t)$$

where *λ*_*k*_, *l*^*k*^, *r*^*k*^, *k *= 1,..., *n *are the eigenvalues, the left eigenvectors (vector-rows) and the right eigenvectors (vector-columns) of the matrix *K*, respectively, i.e.

(3) *K r*^*k *^= *λ*_*k *_*r*^*k*^, *l*^*k *^*K *= *λ*_*k *_*l*^*k*^.

with the normalization (*l*^*i*^, *r*^*j*^) = *δ*_*ij*_, where *δ*_*ij *_is Kronecker's delta.

Closed systems are characterized by *K*_0 _= 0 (no production reactions, although degradation is permitted). Close systems are conservative if the matrix *K *is singular (a particular case is when there is no degradation at all). Then, the left kernel of *K *provides a set of conservation laws (if *l K *= 0, then quantities (*l, c*) are conserved). Solution of the homogeneous linear equations are simply:

(4) $$c(t)=\sum_{k=1}^{n}r^{k}(l^{k},c(0))\exp(-\lambda_{k}t)$$

If all reaction constants *k*_*ij *_would be known with precision then the eigenvalues and the eigenvectors of the kinetic matrix can be easily calculated by standard numerical techniques. Furthermore, singular value decomposition can be used for model reduction. But in systems biology models often one has only approximate or relative values of the constants (information on which constant is bigger or smaller than another one). In the further we will consider the simplest case: when all kinetic constants are very different (separated), i.e. for any two different pairs of indices *I *= (*i*, *j*), *J *= (*i'*, *j'*) we have either *k*_*I *_>> *k*_*J *_or *k*_*J *_>> *k*_*I*_. In this case we say that the system is hierarchical with timescales (inverses of constants *k*_*ij*_, *j *≠ 0) *totally separated*.

**Figure 1:**
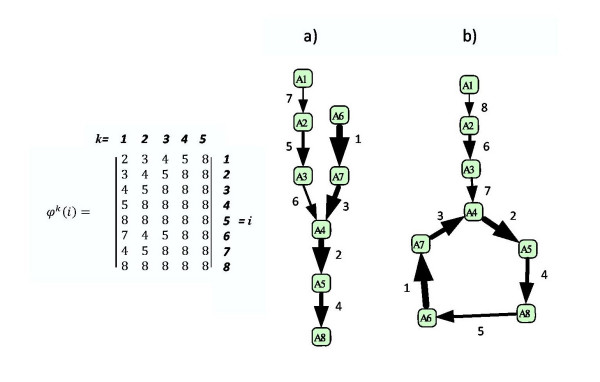
**Two simple examples of exactly solvable linear kinetics.** a) non-branching network without cycles. b) network with a unique sink which is a cycle. On the left, *ϕ*(*i*) map is shown for the network a). The order of kinetics parameters is shown both by integer numbers (ranks) and the thickness of arrows (faster reactions are thicker).

Hierarchical linear network can be represented as a digraph and a set of orders (integer numbers) associated to each arc (reaction). The lower the order, the more rapid is the reaction (see Fig. 1). It happens that in this case the special structure of the matrix *K *(originated from a reaction graph) allows us to exploit the strong relation between the dynamics (1) and the topological properties of the digraph. Big advantage of the fully separated network is that the possible values of $l_{i}^{k}$ are 0, 1 and the possible values of $r_{i}^{k}$ are -1, 0, 1 with high precision [34]. Thus, if we can provide an algorithm for finding non-zero components of $l_{i}^{k}$, $r_{i}^{k}$, based on the network topology and the constants ordering, then this will give us a good approximation to the problem solution (2).

#### Some basic notions

Two vertices of a graph are called adjacent if they share a common edge. A path is a sequence of adjacent vertices. A graph is connected if any two of its vertices are linked by a path. A maximal connected subgraph of graph *G *is called a connected component of *G*. Every graph can be decomposed into connected components.

A directed path is a sequence of adjacent edges where each step goes in direction of an edge. A vertex *A *is *reachable *from a vertex *B*, if there exists an oriented path from *B *to *A*.

A nonempty set *V *of graph vertexes forms a *sink*, if there are no oriented edges from *A*_*i *_∈ *V *to any *A*_*j *_∉ *V*. For example, in the reaction graph *A*_1 _← *A*_2 _→ *A*_3 _the one-vertex sets {*A*_1_} and {*A*_3_} are sinks. A sink is minimal if it does not contain a strictly smaller sink. In the previous example, {*A*_1_}, {*A*_3_} are minimal sinks. Minimal sinks are also called ergodic components.

A digraph is strongly connected, if every vertex *A *is reachable from any other vertex *B*. Ergodic components are maximal strongly connected subgraphs of the graph, but the reverse is not true: there may exist maximal strongly connected subgraphs that have outgoing edges and, therefore, are not sinks. If the digraph has no branching (each vertex has only one successor), then we can define a deterministic flow (discrete dynamical system) on the set of its vertices. Every vertex is the origin of an unique directed path.

#### Basic procedure for approximating eigenvectors

**Figure 2:**
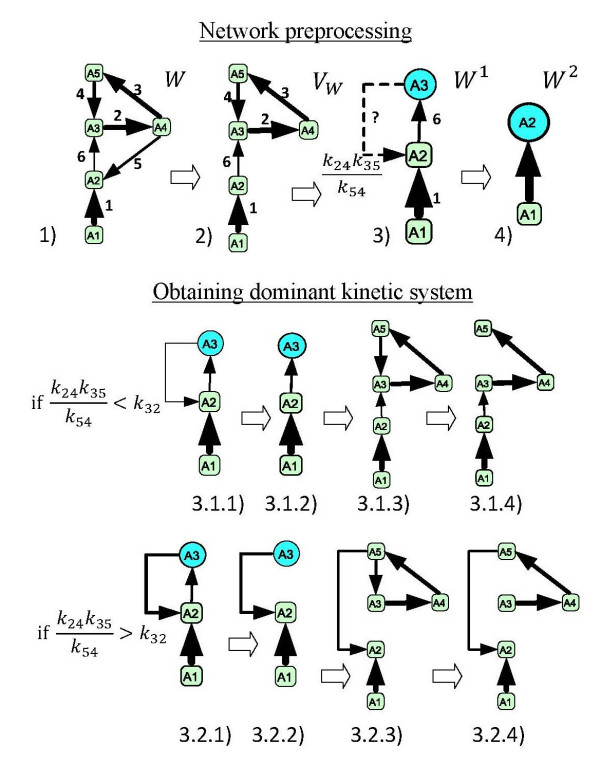
**Example of calculation of the dominant approximation for a linear separated reaction network shown (1).** See the text for the details. The order of kinetics parameters is shown both by integer numbers (ranks) and the thickness of arrows (faster reactions are thicker).

The algorithm we provide is based on the solution of two simplest cases: 1) network without cycles and without branching (i.e, there are no vertices with more than one outgoing edges) (for example, Fig. 1a) and 2) network without branching with a unique sink which is a cycle (for example, Fig. 1b).

For the networks without branching, we can simplify the notation for the kinetic constants, by introducing *κ*_*i *_= *k*_*ij*_. Also it is useful to introduce a map *ϕ *(see Fig. 1):

(5) $$\phi (i)=\{\begin{array}{ll} j, & \text{if there exists }A_{i}\to A_{j}\\ i, & \text{else} \end{array}$$

#### Acyclic non-branching network

In this case, for any vertex *A*_*i *_there exists an eigenvector. If *A*_*i *_is a sink vertex (i.e. *ϕ*(*i*) = *i*) then this eigenvalue is zero. If *A*_*i *_is not a sink (i.e. *ϕ*(*i*) ≠ *i *and reaction *A*_*i *_→ *A*_*ϕ*(*i*) _has nonzero rate constant *κ*_*i*_) then this eigenvector corresponds to eigenvalue -*κ*_*i*_. For left and right eigenvectors of *K *that correspond to *A*_*i *_we use notations *l*^*i *^(vector-row) and *r*^*i *^(vector-column), correspondingly.

Let us suppose that *A*_*f *_is a sink vertex of the network. Its associated right and left eigenvectors corresponding to the zero eigenvalue are given by:

(6) $$\begin{array}{r} r_{j}^{f}=\delta_{j}^{f}\\ l_{j}^{f}=\{\begin{array}{ll} 1, & \text{if }\phi^{q}(f)=j\text{ for some }q>0\\ 0, & \text{else} \end{array} \end{array}$$

Generally, right eigenvectors can be constructed by recurrence starting from the vertex *A*_*i *_and moving in the direction of the flow. The construction is in opposite direction for left eigenvectors.

For right eigenvector *r*^*i *^only coordinates $r_{\phi^{k}(i)}^{i}$ (*k *= 0, 1, ..) can have nonzero values, and

(7) $$\begin{array}{r} r_{\phi^{k+1}(i)}^{i}=\frac{\kappa_{\phi^{k}(i)}}{\kappa_{\phi^{k+1}(i)}-\kappa_{i}}r_{\phi^{k}(i)}^{i}=\prod_{j=0}^{k}\frac{\kappa_{\phi^{j}(i)}}{\kappa_{\phi^{j+1}(i)}-\kappa_{i}}\\ =\frac{\kappa_{i}}{\kappa_{\phi^{k+1}(i)}-\kappa_{i}}\prod_{j=1}^{k}\frac{\kappa_{\phi^{j}(i)}}{\kappa_{\phi^{j}(i)}-\kappa_{i}}. \end{array}$$

For left eigenvector *l*^*i *^coordinate $l_{j}^{i}$ can have nonzero value only if there exists such *q *≥ 0 that *ϕ*^*q *^(*j*) = *i *(this *q *is unique because the system of reactions has no cycles), and

(8) $$l_{j}^{i}=\frac{\kappa_{j}}{\kappa_{j}-\kappa_{i}}l_{\phi (j)}^{i}=\prod_{k=0}^{q-1}\frac{\kappa_{\phi^{k}(j)}}{\kappa_{\phi^{k}(j)}-\kappa_{i}}.$$

These formulas (7, 8) are true for all non-branching acyclic linear systems, even without separation of times. In the case of fully separated systems, they are significantly simplified and do not require knowledge of the exact values of *κ*_*i*_. Thus, for the left eigenvectors $l_{i}^{i}$ = 1 and, for *i *≠ *j*,

(9) $$l_{j}^{i}=\{\begin{array}{ll} 1, & \text{if }\phi^{q}(j)=i\text{ for some }q>0\text{ and }\kappa_{\phi^{d}(i)}>\kappa_{i}\text{ for all }d=0,...q-1\\ 0, & \text{else} \end{array}$$

For the right eigenvectors we suppose that *κ*_*f *_= 0 for a sink vertex *A*_*f*_. Then $r_{i}^{i}$ = 1 and

(10) $$r_{\phi^{k}(j)}^{i}=\{\begin{array}{ll} -1, & \text{if }\kappa_{\phi^{k}(i)}<\kappa_{i}\text{ and }\kappa_{\phi^{m}(i)}>\kappa_{i}\text{ for all }m=1,...k-1\\ 0, & \text{else} \end{array}$$

Vector *r*^*i *^has at most two non-zero coordinates. The formula (10) means that to find the -1 component in *r*^*i*^, one should find the first vertex *j *downstream of *i *with *κ*_*j *_<*κ*_*i *_("bottleneck" vertex): there $r_{j}^{i}$ = -1. Following (10,9) we find that for the example at Fig. 1a

(11) $$\begin{array}{l} l^{1}\approx (1,0,0,0,0,0,0,0),\;l^{2}\approx (0,1,0,0,0,0,0,0),\\ l^{3}\approx (0,1,1,0,0,0,0,0),\;l^{4}\approx (0,0,0,1,0,0,0,0),\\ l^{5}\approx (0,0,0,1,1,1,1,0),\;l^{6}\approx (0,0,0,0,0,1,0,0).\\ l^{7}\approx (0,0,0,0,0,1,1,0) \end{array}$$

(12) $$\begin{array}{l} l^{1}\approx (1,0,0,0,0,0,0,-1),\;l^{2}\approx (0,1,-1,0,0,0,0,0),\\ l^{3}\approx (0,0,1,0,0,0,0,-1),\;l^{4}\approx (0,0,0,1,-1,0,0,0),\\ l^{5}\approx (0,0,0,0,1,0,0,-1),\;l^{6}\approx (0,0,0,0,0,1,-1,0).\\ l^{7}\approx (0,0,0,0,-1,0,1,0) \end{array}$$

#### Non-branching network with a unique simple cyclic sink

In this case we have a reaction network with components *A*_1_, ... *A*_*n *_and last *τ *vertices (after some change of enumeration) form a reaction cycle: *A*_*n*-*τ*+1 _→ *A*_*n*-*τ*+2 _→ ... *A*_*n *_→ *A*_*n*-*τ*+1_. We assume that the limiting step in this cycle (reaction with minimal constant) is *A*_*n *_→ *A*_*n*-*τ*+1_.

In this case the right eigenvector corresponding to the zero eigenvalue has non-zero components only on the vertices belonging to the cycle:

(13) $$l_{j}^{i}=\{\begin{array}{ll} 1, & \text{if }\phi^{q}(j)=i\text{ for some }q>0\text{ and }\kappa_{\phi^{d}(i)}>\kappa_{i}\text{ for all }d=0,...q-1\\ 0, & \text{else} \end{array}$$

Similarly, the stationary distribution has non-zero value only at vertices belonging to the cycle. If *b *= ∑_*i *_*c*_*i *_is the total (conserved) mass, then the steady state is:

(14) $$c_{j}=\frac{b/\kappa_{j}}{\frac{1}{\kappa_{n-\tau +1}}+\frac{1}{\kappa_{n-\tau +2}}+\ldots \frac{1}{\kappa_{n}}}$$

for *j *∈ [*n *- *τ *+ 1, *n*] and zero elsewhere.

If we have a system with well separated constants (which means that *κ*_*n *_≪ *κ*_*i*_, *i *≠ *n*) then this expression in the first order is simplified to

(15) $$\begin{array}{cc} c_{n}=b\left(1-\sum_{i=n-\tau +1}^{n-1}\frac{\kappa_{n}}{\kappa_{i}}\right), & c_{i}=b\frac{\kappa_{n}}{\kappa_{i}} \end{array},$$

which means that most of substance is concentrated just before the "bottleneck" *A*_*n *_→ *A*_*n*-*τ*+1 _(*c*_*n *_≫ *c*_*i*_, *i *≠ *n*).

To approximate the dynamics of the reaction network for *κ*_*n *_≪ *κ*_*i*_, *i *≠ *n*, it is sufficient to remove the slowest step of the cycle *A*_*n *_→ *A*_*n*-*τ*+1_. After removing, we will have acyclic non-branching system of reactions with eigenvalues and eigenvectors that can be computed from the formulas in the previous section. These formulas give *n *- 1 eigenvector sets corresponding to *n *- 1 non-zero eigenvalues *λ*_*i *_= -*κ*_*i*_, *i *= 1..*n *- 1. For example, removing *A*_8 _→ *A*_6 _step at Fig. 1b converts the reaction network to the Fig. 1 a whose dynamics approximates the dynamics of the simple cyclic network.

#### Auxiliary reaction network and auxiliary dynamical system

Now let us consider an arbitrary linear reaction network with well-separated constants. For each *A*_*i*_, let us define *κ*_*i *_as the maximal kinetic constant for reactions *A*_*i *_→ *A*_*j*_: *κ*_*i *_= max_*j*_{*k*_*ji*_}. For correspondent *j *we use notation *ϕ*(*i*): *ϕ*(*i*) = arg max_*j*_{*k*_*ji*_}. The function *ϕ*(*i*) is defined under condition that for *A*_*i *_outgoing reactions *A*_*i *_→ *A*_*j *_exist. If there exist no such outgoing reactions then let us define *ϕ*(*i*) = *i*.

An auxiliary reaction network is the set of reactions *A*_*i *_→ *A*_*ϕ*(*i*) _with kinetic constants *κ*_*i*_. The correspondent kinetic equation is

(16) $${\dot{c}}_{i}=-\kappa_{i}c_{i}+\sum_{\phi (j)=i}\kappa_{j}c_{j},$$

The auxiliary network also defines a auxiliary discrete dynamical system *i *→ Φ (*i*) that is used to compute the eigenvectors of the kinetic matrix. The auxiliary network can have several connected components. In each connected component the minimal sink is an attractor of the auxiliary dynamical system, hence it is either a node, or a cycle.

### General algorithm for calculating the dominant behavior of the linear dynamics

#### Preprocessing reaction network

1) Let us consider a reaction network W with a given structure and fixed ordering of constants that are well separated. Using this ordering let us construct the auxiliary reaction network $V=V_{W}$.

2) If the auxiliary network does not contain cycles then the auxiliary network kinetics (16) approximates relaxation of the initial network W. To obtain the solution, we use directly formulas (7,8) to calculate the eigenvectors (if all *κ*_*i *_are known) or (9,10) to obtain the 0–1 asymptotics (if only the ordering of *κ*_*i *_is known).

3) In general case, let the system V have several cycles *C*_1_, *C*_2_, ... with periods *τ*_1_, *τ*_2_, ... > 1.

By "gluing" cycles into points, we transform the reaction network W into $W^{1}$ as follows. For each cycle *C*_*i*_, we introduce a new vertex *A*^*i*^. The new set of vertices is $A^{1}=A\cup \{A^{1},A^{2},...\}\backslash (\cup_{i}C_{i})$ (we delete cycles *C*_*i *_and add vertices *A*^*i*^).

Let us consider all the reactions from W of the form *A *→ *B *(*A*, *B *∈ A). They can be separated into 5 groups:

1. both *A*, *B *∉ ∪_*i *_*C*_*i*_;

2. *A *∉ ∪_*i *_*C*_*i*_, but *B *∈ *C*_*i*_;

3. *A *∈ *C*_*i*_, but *B *∉ ∪_*i *_*C*_*i*_;

4. *A *∈ *C*_*i*_, *B *∈ *C*_*j*_, *i *≠ *j*;

5. *A*, *B *∈ *C*_*i*_.

1. Reactions from the first group ("transitive" reactions) do not change.

2. Reactions from the second group ("entering to cycles") transform into *A *→ *A*^*i *^(to the whole glued cycle) with the same constant.

3. Reactions of the third type ("exiting from cycles") change into *A*^*i *^→ *B *with the rate constant renormalization: let the cycle *C*^*i *^be the following sequence of reactions *A*_1 _→ *A*_2 _→ ... $A_{\tau_{i}}$ → *A*_1_, and the reaction rate constant for *A*_*i *_→ *A*_*i*+1 _is *k*_*i *_($k_{\tau_{i}}$ for $A_{\tau_{i}}$ → *A*_1_). For the limiting reaction of the cycle *C*_*i *_we use notation *k*_lim *i*_. If *A *= *A*_*j *_and *k *is the rate reaction for *A *→ *B*, then the new reaction *A*^*i *^→ *B *has the rate constant *kk*_lim *i*_/*k*_*j*_. This corresponds to a quasistationary distribution on the cycle (15). It is obvious that the new rate constant is smaller than the initial one: *kk*_lim *i*_/*k*_*j *_<*k*, because *k*_lim *i *_<*k*_*j *_due to definition of limiting constant. If after gluing, several reactions *A*^*i *^→ *B *appear, then only the one with the maximal constant should be kept.

4. The same constant renormalization is necessary for reactions of the fourth type ("between cycles"). These reactions transform into *A*^*i *^→ *A*^*j*^.

5. Reactions of the fifth type ("inside cycles") are discarded.

4) After the new network $W^{1}$ is constructed, we assign $W:=W^{1}$, $A:=A^{1}$ and iterate the algorithm from the step 1) until we obtain an acyclic network and exit at step 2).

The algorithm produces an hierarchy of cycles. Notice that the algorithm is based on an asymmetry between entering reactions and outgoing reactions from cycles in the hierarchy. Indeed, some fluxes of W entering cycles *C*_*i *_can be neglected when they are dominated by a stronger flux of V bifurcating from the same node (this occurs at the first step of the algorithm when constructing V). The cycles *C*_*i *_are minimal sinks in V (they are attractors of the auxiliary dynamical system). There are no reactions *A *→ *B *in V such that *A *∈ *C*_*i*_, *B *∉ *C*_*i*_. Nevertheless, there may be such reactions in the initial network W. These fluxes can not be neglected because there are no exiting fluxes of V to dominate them. The rule of thumb is: neglect any dominated flux except for the fluxes exiting some cycle in the hierarchy. This explains our algorithm and was rigorously justified in [34].

#### Constructing the dominant kinetic system

Now we show how to find an approximation of the dynamics of the reaction network W. To construct this approximation, we produce a new acyclic reaction network with the initial set of vertices *A*_*i *_∈ W, *i *= 1..*n *which is called *dominant kinetic system*. Dynamics of this acyclic system can be computed from (7,8,9,10). To construct the dominant kinetic system, the following algorithm is applied:

Let $V^{m}$ be the result of the network preprocessing algorithm described in the previous section.

1. For $V^{m}$ let us select the vertices $A_{1}^{m}$, $A_{2}^{m}$, ... that are glued cycles from $V^{m-1}$.

2. For each glued cycle node $A_{i}^{m}$:

a) Recall its nodes $A_{i1}^{m-1}\to A_{i2}^{m-1}\to \ldots A_{i\tau_{i}}^{m-1}\to A_{i1}^{m-1}$; they form a cycle of length *τ*_*i*_,

b) Let us assume that the limiting step in $A_{i}^{m}$ is $A_{i\tau_{i}}^{m-1}\to A_{i1}^{m-1}$,

c) Remove $A_{i}^{m}$ from $V^{m}$,

d) Add *τ*_*i *_vertices $A_{i1}^{m-1},A_{i2}^{m-1},\ldots A_{i\tau_{i}}^{m-1}$ to $V^{m}$,

e) Add to $V^{m}$ reactions $A_{i1}^{m-1}\to A_{i2}^{m-1}\to \ldots A_{i\tau_{i}}^{m-1}$ (that are the cycle reactions without the limiting step) with correspondent constants from $V^{m-1}$,

f) If there exists an outgoing reaction $A_{i}^{m}$ → *B *in $V^{m}$ then we substitute it by the reaction $A_{i\tau_{i}}^{m-1}$ → *B *with the same constant, i.e. outgoing reactions $A_{i}^{m}$ → ... are reattached to the heads of the limiting steps,

g) If there exists an incoming reaction in the form *B *→ $A_{i}^{m}$, find its prototype in $V^{m-1}$ restore it in $V^{m}$.

3. If in the initial $V^{m}$ there existed a "between-cycles" reaction $A_{i}^{m}\to A_{j}^{m}$ then we find the prototype in $V^{m-1}$, *A *→ *B*, and substitute the reaction by $A_{i\tau_{i}}^{m-1}$ → *B *with the same constant, as for $A_{i}^{m}\to A_{j}^{m}$ (again, the beginning of the arrow is reattached to the head of the limiting step in $A_{i}^{m}$).

4. Let *m *← *m *- 1, and repeat steps 1–4 until no glued cycles left.

One has to notice that in the process of network preprocessing some reaction rates are substituted by monomials of the initial reaction constants, i.e. expressions in the form $k_{new}=\frac{k_{i_{1},i_{2}}...k_{j_{1},j_{2}}}{k_{l_{1},l_{2}}...k_{m_{1}}{,}_{m_{2}}}$. In totally separated case the values of these monomials are also well separated from the other constants with probability close to 1, however, the initial order of constants does not prescribe position of these monomials in the rates order. In this case the algorithm produces several dominant systems defined for all possible position of new monomial rate constants in the order. An example of this will be given later in this section. Such a situation can happen during the network preprocessing when maximum reaction constant should be chosen, or in the process of dominant system creation, when determining the limiting step in a cycle.

#### Finding stationary distributions

The dominant kinetic system fully describes the relaxation modes of the network. The construction of this system depends only on the matrix *K *and does not depend on the production reactions *K*_0_. In particular, relaxation times do not depend on the system being closed or not. However, the stationary distribution *c*^*s *^and the sequence of relaxation events depends on production reactions (see Eq.(2)).

For closed systems, steady states are solutions of the linear homogeneous equation *Kc *= 0, therefore they are determined up to multiplication by positive constants. They form a *k*-dimensional cone where *k *is the multiplicity of the zero eigenvalue of the matrix *K*, also the number of minimal sinks of the network.

Let $A_{f1}^{m},A_{f2}^{m},...,A_{fk}^{m}$ be *k *sink vertices of the auxiliary network $V^{m}$. Let *A*_*i*_, *i *= 1..*n *be vertices in the initial network W. Below we describe a procedure for finding the basis of all stationary distributions of a closed network:

1. Let us take the *i*th sink vertex $A_{fi}^{m}$.

2. Define *x *= $A_{fi}^{m}$, *b *= 1, and a null vector *b*^*i *^∈ *R*^*n*^.

3. If *x *is not a glued cycle then it corresponds to a vertex *A*_*j *_∈ W, and the basis vector *b*^*i *^has components $b_{j}^{i}$ = *δ*_*ij*_; stop.

4. If *x *is a glued cycle then recall all its vertices *x*^1^, ..., *x*^*τ*^.

5. Determine the limiting (minimal) rate constant *κ*_*lim *_= min_*s*=1..*τ *_{$\kappa_{x^{s}}$} and *s*_*min *_= arg min_*s*=1..*τ *_{$\kappa_{x^{s}}$}.

6. For each vertex *x*^*j *^of the cycle repeat:

a) Let $c_{j}=b\frac{\kappa_{lim}}{\kappa_{x^{j}}}$ if *j *≠ *s*_*min *_and $c_{j}=b\{1-\sum_{s\neq s_{min}}\frac{\kappa_{lim}}{\kappa_{x^{s}}}\}$ otherwise,

b) if *x*^*j *^corresponds to a simple vertex *A*_*k *_then $b_{k}^{i}$ = *c*_*j*_,

c) if *x*^*j *^corresponds to a glued cycle then do recursively steps 4–6 with *x *= *x*^*j *^and *b *= *c*_*j*_.

Any possible stationary distribution has form $c^{s}=\sum_{i=1..k}c_{fi}^{m}b^{i},c_{fi}^{m}\geq 0$, $c_{fi}^{m}$. The coefficients ${\tilde{c}}^{s}=\sum_{i=1..k}c_{fi}^{m}{\tilde{b}}^{i}$ are computed from initial data: they are equal to the total initial mass carried by vertices of $V^{m}$ (when these are glued cycles we consider the total initial mass of the cycle) that are attracted by $A_{fi}^{m}$.

In brief, the distribution of the concentrations on any cycle is approximated by the first order expression (15), and this procedure is applied recursively for the vertices that represent glued cycles. The state thus obtained is equally a good approximation of the steady state of the dominant kinetic system.

Open systems can be reduced to closed ones by considering that all production reactions originate from the node ∅ that has concentration *c*_0 _= 1. The corresponding reaction are ∅ → *A*_*i *_and the constants are the production rates *k*_*i*0_. The normalization *c*_0 _= 1 is possible for all bounded steady states, because these are determined up to multiplication by a constant. Furthermore, all steady states are bounded, provided that the following topological condition holds: if there exists an oriented path from ∅ to *A*_*i*_, then there exists an oriented path from *A*_*i *_to ∅. We suppose this condition to be always fulfilled. Applying the algorithm to the closed system we obtain ${\tilde{c}}^{s}={\tilde{c}}^{s}/(\sum_{i=1..k}c_{fi}^{m}{\tilde{b}}_{0}^{i})$ that is normalized to ${\tilde{c}}^{s}={\tilde{c}}^{s}/(\sum_{i=1..k}c_{fi}^{m}{\tilde{b}}_{0}^{i})$ in order to have $c_{0}^{s}$ = 1.

**Example**. Let us consider an example of the network W shown on Fig. 2 (1). Below we briefly detail every step of the algorithm.

2) An auxiliary reaction network $V_{W}$ is constructed (this gives a non-branching network);

3) The cycle *A*_3 _→ *A*_4 _→ *A*_5 _→ *A*_3 _in the auxiliary reaction network is glued in one vertex $A_{3}^{1}$ (shown by the circle node); In the initial network we find an "exiting from cycle" reaction *A*_4 _→ *A*_2_, renormalize its rate to $k_{32}^{1}=\frac{k_{24}k_{35}}{k_{54}}$ and insert in the new network $W^{1}$;

4) The cycle *A*_3 _→ *A*_2 _→ *A*_3 _in the auxiliary reaction network $V_{W^{1}}$ (which is now coincides with $W^{1}$ itself) is glued in one vertex $A_{2}^{2}$; now the network $W^{2}$ is acyclic and we stop the network preprocessing.

Now if we restore the cycle *A*_3 _→ *A*_2 _→ *A*_3 _and try to determine the limiting step in it, we have two possibilies: $\frac{k_{24}k_{35}}{k_{54}}$ <*k*_32 _and $\frac{k_{24}k_{35}}{k_{54}}$ > *k*_32_. Let us consider them separately:

*Case *$\frac{k_{24}k_{35}}{k_{54}}$ <*k*_32_

3.1.1) Since $A_{3}^{1}$ <*k*_32_, then we remove the limiting step $A_{3}^{1}$ → *A*_2 _and obtain 3.1.2).

3.1.3) We restore the glued cycle corresponding to $A_{3}^{1}$ and we recall that the reaction *A*_2 _→ $A_{3}^{1}$ in $W^{1}$ corresponds to *A*_2 _→ *A*_3 _in W.

3.1.4) We remove the limiting step reaction in the cycle *A*_3 _→ *A*_4 _→ *A*_5 _→ *A*_3 _(it is *A*_5 _→ *A*_3_) and as a result we obtain acyclic dominant kinetic system shown at Fig. 2 (3.1.4).

*Case *$\frac{k_{24}k_{35}}{k_{54}}$ > *k*_32_

3.2.1) Since $\frac{k_{24}k_{35}}{k_{54}}$ > *k*_32_, then we remove the limiting step *A*_2 _→ $A_{3}^{1}$ and obtain 3.1.2).

3.2.3) We restore the glued cycle corresponding to $A_{3}^{1}$ this time we should re-attach the reaction $A_{3}^{1}$ → *A*_2 _to the head of the limiting step in the cycle (it is *A*_5 _vertex); the rate of *A*_5 _→ *A*_2 _is $\frac{k_{24}k_{35}}{k_{54}}$.

3.2.4) We remove the limiting step reaction in the cycle *A*_3 _→ *A*_4 _→ *A*_5 _→ *A*_3 _(it is *A*_5 _→ *A*_3_) and as a result we obtain acyclic dominant kinetic system shown at Fig. 2 (3.2.4).

### Discussion and perspectives

Dominant approximations of hierarchical linear reaction network allow us to introduce some new concepts important for the dynamics of multiscale systems.

#### Hybrid and qualitative dynamics

Piecewise affine dynamics has been widely used to approximate dynamics of gene regulatory networks [35-37] as a sequence of discrete transitions between attractors of affine systems. This picture is based on threshold response of genes in models with steep regulation functions (Hill functions and other representations of sigmoidal response) and is not directly related to time scales. Here, we emphasize another possible way to obtain hybrid, or qualitative representations of dynamics, based on time separation.

Indeed, zero-one approximation of eigenvectors in hierarchical linear systems justifies a discrete coding of dynamics. Suppose that initial state is concentrated in *j*_0_, *c*_*i *_(0) ~ $\delta_{i,j_{0}}$. At times just larger than 1/*λ*_*k *_an exponential vanishes in Eq. (2) and the state has a "jump" -*r*^*k *^(*l*^*k*^, *c*(0) -*c*_*s*_). Let us consider that eigenvalues are ordered *λ*_1 _>> *λ*_2 _>> ... >> *λ*_*n*-1_. Then, the sequence of right eigenvectors *r*^*k *^such that (*l*^*k*^, *c*(0) -*c*_*s*_) ≠ 0 codes the dynamics starting in *c*(0). In other words, there is a sequence of well separated times *τ*_1 _= 1/*λ*_1 _<<*λ*_2 _= 1/*λ*_2 _<< ... <<*τ*_*n*-1 _= 1/*λ*_*n*-1 _such that something happens (a state transition) between each one of these times. Left eigenvectors provides the lumping (several species cumulated to form pseudospecies) and right eigenvectors provide the sequence of state transitions. On timescales *τ*_*k *_<*t *<*τ*_*k*+1 _one can observe a jump -*r*^*k *^in state space provided that (*l*^*k*^, *c*(0) - *c*^*s*^) ≠ 0. On this timescale the dynamics is equivalent to the degradation of pseudospecies (*l*^*k*^, *c*), $\frac{d(l^{k},c)}{dt}$ = -*λ*_*k *_(*l*^*k*^, *c*).

#### Critical parameters and design principles

Our approach to dominant subsystems emphasizes some simple but important principles. First of all, dynamics of a hierarchical linear network can be specified if a) the topology of the network is given and if b) to each reaction we associate a positive integer representing order (1 for the most rapid reaction, 2 for the second most rapid reaction, and so on ...); c) for cyclic topologies, some monomials grouping constants of several reactions have also to be ordered in the same manner (which reactions depend both on topology and on initial ordering).

In the process of simplification some reaction pathways are dominated and do not appear in the dominant subsystem. Therefore, the corresponding constants are not critical for the system: although their ordering matters for establishing the simplification, their precise value have little importance. Because parameters of the dominant subsystem are generally monomials of parameters of the whole system, critical parameters are those parameters that occur in critical monomials. Our findings show rather counter-intuitive properties of critical and non-critical parameters, that can be useful as design principles. Thus, in cycles, the limiting step (slowest reaction) has little influence on dynamics (though is important for the steady state). Dynamically, a cycle with separated constants behaves like the chain obtained from the cycle by eliminating the limiting step. In particular, the slowest relaxation time of a cycle is the inverse of its *second *slowest constant [1,34].

**Figure 3:**
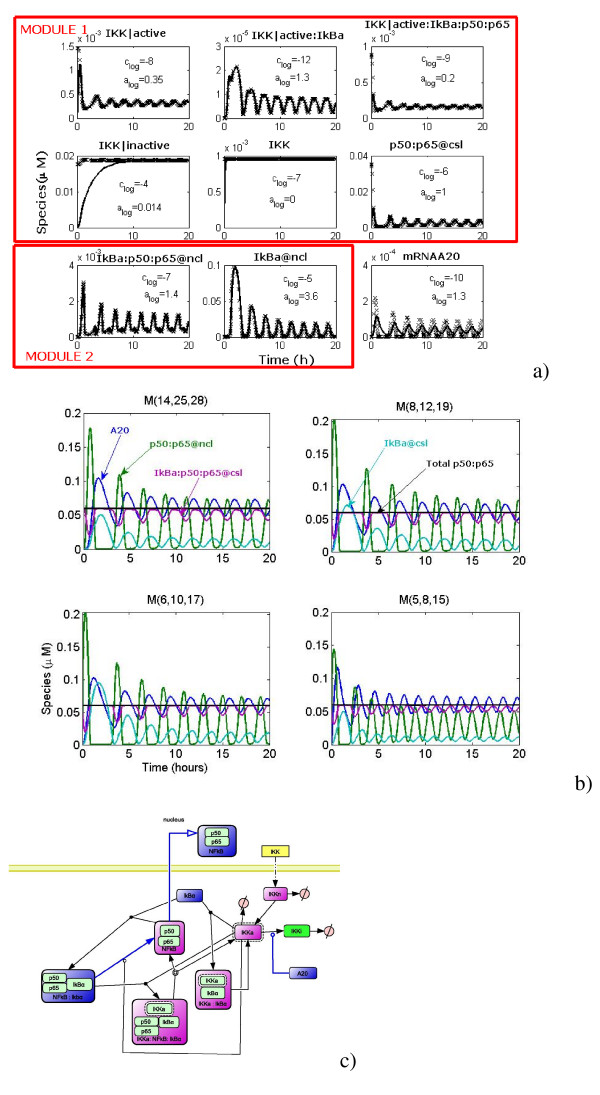
**Lipniacki's model a) Testing quasistationarity: nonreduced trajectories (solid), quasi-stationarity trajectories (crosses).** b) Trajectories of models in the hierarchy. c) Cytoplasmatic part of the signalling mechanism: terminal species (blue), intermediate species quasi-stationary (pink) non-oscillating (green), simple submechanisms (blue). This part of the network contains three critical parameters for the damping time. Sustained oscillations were obtained by decreasing the constant *k*_3 _ten times with respect to the value used in [53] (equivalently, this can be obtained by decreasing *k*_9_, or by increasing *k*_4_).

We should add some words about the relation between linear and non-linear models. Mathematical models of biochemical reaction networks in molecular biology contain with necessity non-linear, non-monomolecular reactions (complex binding, catalysis, etc.). However, the developed algorithms of model reduction for linear networks can be useful in systems biology, in several situations:

1) *When some submechanisms of a complex and non-linear network are linear*, given fixed (or slowly changing) values of external inputs (boundaries);

2) *For approximating non-linear dynamics*. For multiscale nonlinear reaction networks the expected dynamical behaviour is to be approximated by the system of dominant networks. These networks may change in time but remain small enough. To give an example, we provided the Fig. 3S1–3S2 in Additional File 1 demonstrating that in a model of complex reaction network of NF-*κ*B pathway, containing 17 multimolecular reactions, only two reactions show genuinely non-linear behavior in some windows of time, with two more showing border-line behavior, and all others have well-separated reactant concentrations in any moment of time. The rigorous justification of these hybrid approximations for mass action reaction networks will be discussed elsewhere.

### Reduction of non-linear multiscale systems

Complex formation is a source of nonlinearity in biochemical networks. For instance, in signalling, ligand molecules form complexes with receptors. Transcription factors are often dimers or multimers or are sequestered by forming complexes with their inhibitors. In these examples, the reaction rates are non-linear functions of the concentrations of two or more molecules.

To construct a nonlinear reaction network we need the list of components, A = {*A*_1_, ..., *A*_*n*_} and the list of reactions (the reaction mechanism):

(17) $$\sum_{i}\alpha_{ji}A_{i}\to \sum_{k}\beta_{jk}A_{k},$$

where *j *∈ [1, *r*] is the reaction number. Unless reactants and products belong to compartments of different volumes *α*_*ji*_, *β*_*jk *_are nonnegative integers (stoichiometric coefficients). Reactions involving components from different compartments have non-integer stoichiometry. For instance, a reaction translocating a molecule from nucleus to the cytosol has stoichiometry (..., 1, *k*_*v*_, ...) where *k*_*v *_is the volume ratio of cytosol to nucleus.

Dynamics of nonlinear networks is described by a system of differential equations:

(18) $$\frac{dc}{dt}=F(c)=\sum_{j=1}^{r}\nu_{j}R_{j}(c)=SR(c)=\sum_{j=1}^{r}\nu_{j}(R_{j}^{+}(c)-R_{j}^{-}(c))$$

*ν*_*j *_= *β*_*j *_- *α*_*j *_is the global stoichiometric vector. *S *is the stoichiometric matrix whose columns are the vectors *ν*_*j*_. The reaction rates $R_{j}^{+/-}$ (*c*) are non-linear functions of the concentrations. For instance the mass action law reads $R_{j}^{+}(c)=k_{j}^{+}\prod_{i}c_{i}^{\alpha_{ji}},R_{j}^{-}(c)=k_{j}^{-}\prod_{i}c_{i}^{\beta_{ji}}$.

There are no simple rules to relate timescales to reaction constants of nonlinear models. The units of the inverse constants of bimolecular reactions are concentration multiplied by time and one needs at least one concentration value in order to construct a timescale. Generally, timescales are functions of many reaction constants and concentration variables. These functions are not necessarily smooth. Near bifurcations (for instance, near Hopf or saddle-node bifurcations), at least one timescale of the system diverges for finite changes of the reaction constants. However, nonlinear biochemical networks have wide distributions of time-scales, as can be shown by simple (Jacobian based) analysis of models.

Various reduction methods of nonlinear models are based on projection of the dynamics on a lower dimensional invariant manifold [4-8]. The reduced models are systems of differential equations, but no longer networks of chemical reactions. Quasi-equilibrium and quasi-stationarity methods keep the network structure of the model and propose lumped reaction mechanisms as dominant subsystems. This approach has some advantages. Indeed, it leads to more transparent analysis of the results and of design principles, produces hierarchies of models and facilitates model comparison. Graphical reduction methods using elementary modes, were proposed by Clarke [26] for chemical systems and more recently in systems biology by Klamt [38]. Similar methods can be found in [39], from which we have borrowed the terminology. The choice of the species to be eliminated and of the reactions to be aggregated, as well as the calculation of rates of elementary modes have no theoretical justification in these methods and their inappropriate use can alter dynamics (for instance, as Clarke noticed, the stability of limit cycles is not guaranteed). Thus, in order to have a complete practical recipe that applies to multiscale biochemical networks we need to solve three more problems: detection of rapid species, resolution of quasi-stationarity equations and calculation of reaction rates of the dominant mechanisms.

A major improvement in calculating dominant subsystems can be obtained by combining quasi-stationarity and averaging. Averaging techniques are widely used in physics and chemistry to simplify models by eliminating fast, oscillating (microscopic) variables [22-24]. Our use of averaging is different, because we employ it to obtain averaged stationarity equations for slow, non-oscillating variables and to eliminate these species. After choosing a "middle" time scale (corresponding to the time resolution of the experiment), we want to reduce all scales that are faster but also all scales that are slower than this middle scale. In order to do that we provide a unified framework for species elimination and reaction aggregation, either by quasi-stationarity (fast species) or by averaged stationarity (slow species).

Let *I *be the set of indices of intermediate components, that will be eliminated. $R^{\left(I\right)}$ is the set of reactions that either produce or consume species from *I*. Rates of $R^{\left(I\right)}$ depend on the concentrations of intermediate species and also on the concentrations of other species, which in the terminology of Temkin [39] are called terminal. Let *T *be the set of indices of terminal species. Terminal species represent the *frontier *between the rest of the system and the subsystems made of intermediate species. Although instead of terminal the name boundary species could be more appropriate, the latter term has already been employed in systems biology with a different meaning, which is species whose concentrations are fixed in a simulation.

Extracting from the matrix *S *the columns corresponding to the reactions $R_{I}$ and the lines corresponding to the species I and T we obtain the intermediate stoichiometric matrix *S*_*I *_and the terminal stoichiometric matrix *S*_*T*_, respectively.

#### Eliminating fast species: quasi-stationarity

In multiscale biochemical systems, some components react much more rapidly to changes in the environment than others. The reasons for the existence of such fast species can be multiple. Thus, rapidly transformed or rapidly consumed molecules (for instance those taking part in metabolic chains or rapid chemical transformations such as phosphorylation), or promoter sites submitted to rapid binding/unbinding processes are examples of fast species. Fast species are good candidates for intermediate species. Indeed, it is easy to prove that they can be eliminated by quasistationarity. When production rates are not weak, fast species are those whose concentrations are small and well separated from the concentrations of other species. Though straightforward, the precise condition connecting quasi-stationarity and smallness of concentrations can not be easily found in literature, hence we briefly discuss it below.

Let *ϵ *be a small parameter, representing concentrations. Suppose for simplicity that reactions $R^{\left(I\right)}$ are pseudo-monomolecular. This means that *S*_*I *_*R*_*I *_(*c*_*I*_, *c*_*T*_) = *K*_*I *_(*c*_*T*_) *c*_*I *_+ $K_{I}^{0}$(*c*_*T*_), where *R*_*I *_is the restriction of the vector *R *to the intermediate species. An important assumption is $K_{I}^{0}$(*c*_*T*_) = O(1) meaning that the production of intermediate species is not weak.

Suppose that among the reactions $R^{\left(I\right)}$ consuming intermediates, at least some have rates of order O(1). This is current, because these reactions produce terminal species which have larger concentrations.

Because *c*_*I *_= O(*ϵ*), it means that *K*_*I *_(*c*_*T*_) = O(1/*ϵ*). This leads to the following asymptotic:

(19) $$\epsilon \frac{d{\tilde{c}}_{I}}{dt}={\tilde{K}}_{I}(c_{T}){\tilde{c}}_{I}+K_{I}^{0}(c_{T})$$

(20) $$\frac{dc_{I^{c}}}{dt}=g(\epsilon {\tilde{c}}_{I},c_{I^{c}})$$

where ${\tilde{c}}_{I}$ = *c*_*I*_/*ϵ*, ${\tilde{K}}_{I}$ = *ϵK*_*I *_= O(1), *I*^*c *^is the complement of *I *designating species other that *I*. Intermediate species are fast and the system (19) can be reduced using Tikhonov's [40,41] and Fenichel's [42] results. According to these results, after a short laps of time, the system evolves on an *invariant manifold *(an invariant manifold is defined by the property that any trajectory starting in the manifold stays inside the manifold) which is at distance O(*ϵ*) from the quasi-steady state (QSS) manifold defined by the quasi-stationarity equations:

(21) $${\tilde{K}}_{I}(c_{T}){\tilde{c}}_{I}+K_{I}^{0}(c_{T})=0$$

Quasi-stationarity equations can be used to express concentrations of the intermediate species as functions of the concentrations of terminal species. If matrix *K*_*I *_has not full rank, conservation laws should be added to the quasi-stationarity equations in order to obtain a full rank system. Let *μ*_1_, ..., *μ*_*k *_be a basis of the left kernel of *S*_*I *_(a complete set of conservation laws). We say that species of indices *I *are quasi-stationary if they approximately fulfill the equations:

(22) *S*_*I *_*R*_*I *_(*c*_*I*_, *c*_*T*_) = 0

and exactly fulfill the conservation laws:

(23) *μ*_*i *_*i*_*I *_= *C*_*i*_,   *i *= 1,..., *k*,

where *C*_*i *_are real constants.

Fast, quasi-stationary species are generally difficult to detect. For instance, the strong production condition $K_{I}^{0}$(*c*_*T*_) = O(1), although informative for understanding of the dynamics, can not be used in practice. Furthermore, small concentration is not a necessary condition for quasi-stationarity. Therefore, our practical method for detection of fast, quasi-stationary species is based on the direct checking of Eqs.(22), (23) (see Fig 3a and the Results section for an example).

Once quasi-stationary species are detected, the recipe proposed by Clarke [26] can be applied to simplify the reaction mechanism. Let us reformulate this recipe here:

1. Eliminate the intermediate concentrations by solving the equations (22), (23). Express *c*_*I *_as function of *c*_*T*_.

2. Replace the mechanism $R^{\left(I\right)}$ by "simple sub-mechanisms".

3. Compute the rates of the simple sub-mechanisms as functions of *c*_*T*_.

The simplicity criterion employed by Clarke does not follow from a physical principle. Nevertheless, in systems biology, biochemical reactions are simplified representations of complex physico-chemical processes. In the absence of detailed information, simplicity arguments are often employed. Elementary modes analysis widely used in metabolic control and gene network analysis [43-45] is based on exactly the same argument.

The same recipe applies also to model comparison, when we want to compare two models which differ in complexity (some species in one model are not present in the second). In this situation we declare the extra species intermediate and apply the three steps of the algorithm.

#### Simple sub-mechanisms and rates

Let us introduce some more definitions. A reaction route is a combination of reactions in $R_{I}$ transforming terminal species into other terminal species and conserving the intermediate species. It is defined by a integer coefficient vector *γ *∈ ℤ^*s*^ (the dimension *s *is the number of reactions in $R_{I}$) satisfying the following three conditions:

(24) *S*_*I *_*γ *= 0

(25) *γ*_*i *_≥ 0,   if the reaction   *i*   is irreversible

(26) ||*S*_*T *_*γ*|| > 0

Reaction routes are usually defined [39] without the condition (26). By imposing this condition, we exclude internal cycles with zero terminal stoichiometry.

A sub-mechanism *M*(*γ*) is the set of all the reactions in the reaction route *γ*, *M*(*γ*) = {*i*|*γ*_*i *_≠ 0}. A sub-mechanism is simple if it is minimal with respect to inclusion, i.e. if *M *(*γ'*) ⊂ *M *(*γ*) ⇒ *γ *= *γ'*. Simple sub-mechanisms are pathways with a minimal number of reactions, connecting terminal species without producing accumulation or depletion of the intermediate species. Thus, they are candidates for reduced reaction mechanisms. Simple sub-mechanisms are minimal dependent sets in oriented matroids [46], similar to elementary modes in flux balance analysis [43]. Algorithms for finding elementary modes can be applied for the search of simple sub-mechanisms [43-45].

In the reduced model, the reactions of the intermediate mechanism $R_{I}$ are replaced by the sub-mechanisms *γ*_1_, ..., *γ*_*s*_.

Each terminal species is produced or consumed by one or several reactions of the intermediate mechanism. The reduction should preserve the flux of each terminal species, meaning that the following equation should be satisfied identically, for all *c*_*T *_and *c*_*I *_satisfying (22),(23)^:^

(27) $$\begin{array}{cc} \sum_{m\in R_{I}}\nu_{m}^{j}R_{m}(c_{I},c_{T})=\sum_{i=1}^{s}{\left(S_{T}\gamma_{i}\right)}_{j}{R^{\prime }}_{i}(c), & j\in T \end{array}$$

where ${R^{\prime }}_{i}(c)$ are the rates of the simple sub-mechanisms.

Suppose that for any simple sub-mechanism *i *there is a terminal species *j *such that *S*_*T *_*γ*_*i *_is the unique vector (among the *s *different ones) having nonzero coordinate *j*, (*S*_*T *_*γ*_*i*_)_*j *_≠ 0. Then, there is a straightforward solution for (27):

(28) $${R^{\prime }}_{i}(c)=\frac{1}{{\left(S_{T}\gamma_{i}\right)}_{j}}\sum_{m\in R_{I}}\nu_{m}^{i}R_{m}(c_{I},c_{T})$$

The above uniqueness condition is not fulfilled if there are two sub-mechanisms for which the terminal stoichiometries are proportional. This situation can be avoided by quotienting with respect to the following equivalence relation: *γ*_*i *_and *γ*_*j *_are equivalent iff *S*_*T *_*γ*_*i *_= *α S*_*T *_*γ*_*j*_, for some *α *= ≠ 0. After discarding some sub-mechanisms and keeping only one representative per class, we have a reduced set of simple sub-mechanisms for which rates can be calculated from (28).

#### Dominant solutions to the quasi-stationarity equations, multiscale ensembles

The most difficult part of the above algorithm is to solve the quasi-stationarity equations (22),(23). Even in the monomolecular case, symbolic solutions of the linear system (21) can involve long expressions. Furthermore, mass action law leads to polynomial equations in the binary or multi-molecular case. Symbolic methods for solutions of systems of polynomial equations are limited to a small number of variables.

In this subsection we show how the multi-scale nature of the system can be used to obtain approximate, dominant solutions of the quasi-stationarity equations.

In linear hierarchical models, ensembles with well separated constants appear (see also [1]). We could represent them by a log-uniform distribution in a sufficiently big interval log *k *∈ [*α*, *β*], but most of the properties of this probability distribution will not be used here. The only property that we will use is the following: if *k*_*i *_> *k*_*j*_, then *k*_*i*_/*k*_*j *_≫ 1 (with probability close to one). It means that we can assume that *k*_*i*_/*k*_*j *_≫ *a *for any preassigned positive value of *a *that does not depend on *k *values. One can interpret this property as an asymptotic one for *α *→ -∞, *β *→ ∞. This property allows us to simplify algebraic formulas. For example, *k*_*i *_+ *k*_*j*_can be substituted by max{*k*_*i*_, *k*_*j*_} (with small relative error), or

$$\frac{ak_{i}+bk_{j}}{ck_{i}+dk_{j}}\approx \{\begin{array}{cc} a/c, & \text{if }k_{i}\gg k_{j};\\ b/d, & \text{if }k_{i}\ll k_{j}, \end{array}$$

for nonzero *a*, *b*, *c*, *d*.

Of course, some ambiguity can be introduced, for example, what is (*k*_1 _+ *k*_2_) - *k*_1_, if *k*_1 _≫ *k*_2_? If we first simplify the expression in brackets, it is zero, but if we open brackets without simplification, it is *k*_2_. This is a standard difficulty in use of relative errors for round-off. If we estimate the error in the final answer, and then simplify, we shall avoid this difficulty. Use of *o *and O symbols also helps to control the error qualitatively: if *k*_1 _≫ *k*_2_, then we can write (*k*_1 _+ *k*_2_) = *k*_1_(1 + *o*(1)), and *k*_1_(1 + *o*(1) - *k*_1 _= *k*_1_*o*(1). The last expression is neither zero, nor absolutely small – it is just relatively small with respect to *k*_1_.

It is slightly more difficult to solve equations. Some recipes were proposed such as Newton polyhedra for approximate solutions of polynomial systems of equations [47] but this type of methods suffers from combinatorial complexity. Here we use a simpler, but not so rigorous approach. In the case of pseudo-molecular subsystems, our algorithms for linear hierarchical systems are enough for this purpose. In general, we choose the dominant terms in the solutions as monomials of the parameters. This can be done either by educated guess, or by testing numerically the orders of various terms in the equations. The most frequent, truly non-linear simplification that occurs in biochemical models is the "min-funnel", which we present below.

Let us consider the production of a complex C from two proteins A and $\text{B}:\emptyset \overset{k_{A}}{\underset{}{\to }}\text{A}$ (production of A), $\emptyset \overset{k_{B}}{\underset{}{\to }}\text{B}$ (production of B), $\text{A}\overset{k_{deg,A}}{\underset{}{\to }}\emptyset$ (degradation of A), $\text{B}\overset{k_{deg,B}}{\underset{}{\to }}\emptyset$ (degradation of B), $\text{A}+\text{B}\overset{k_{c}}{\underset{}{⇌}}\text{C}$ (complex formation).

Supposing A, B quasi-stationary we have to find the positive solutions of the equations $k_{A}=\tilde{A}+{\tilde{k}}_{c}\tilde{A}\tilde{B}$, $k_{B}=\tilde{B}+{\tilde{k}}_{c}\tilde{A}\tilde{B}$, where $\tilde{A}=k_{deg,A}A$, $\tilde{B}=k_{deg,B}B$, ${\tilde{k}}_{c}=k_{c}/(k_{deg,A}k_{deg,B})$. We will consider two cases a) 1/${\tilde{k}}_{c}$ <<*k*_*A *_<<*k*_*B *_and b) 1/${\tilde{k}}_{c}$ <<*k*_*B *_<<*k*_*A*_. Both cases mean that degradation of *A*, *B *is weak and/or the propensity of complex formation is high. Case a) means also that *B *is in excess, the opposite being true in case b).

Let us consider the case a). We consider that the order of $\tilde{A}$ in the dominant solution is larger than the order of $\tilde{B}$, $\tilde{A}<<\tilde{B}$. From the linear equation *k*_*A *_- *k*_*B *_= $\tilde{A}-\tilde{B}$ we obtain $\tilde{B}$ = *k*_*B *_and from the second nonlinear equation we obtain $\tilde{A}=\frac{k_{A}}{1+{\tilde{k}}_{c}k_{B}}\approx \frac{k_{A}}{{\tilde{k}}_{c}k_{B}}$. Finally, we have $\tilde{A}<<\tilde{B}$ consistently with the starting guess. The dominant solution in case b) is obtained by symmetry from the one in case a). The quantity of interest in this example, for which we want a reduced expression is the production rate of the complex *R*_*c *_= *k*_*c*_*AB*. Actually, the two solutions can be summarized by:

(29) *R*_*c *_= *min*(*k*_*A*_, *k*_*B*_)

Using the exact solution of the system (after eliminating *A *from the linear equation we remain with a quadratic equation for *B*) we can show that the min-funnel approximation (29) is valid under less restrictive conditions. The only separation condition that we need is *min*(*k*_*A*_, *k*_*B*_) >> *k*_*deg, A *_*k*_*deg*,*B*_/*k*_*c*_. We can easily identify the critical parameters *k*_*A*_, *k*_*B *_and the non-critical ones *k*_*deg*,*A*_, *k*_*deg*,*B*_, *k*_*c*_. The validity of the expression (29) depends on order relations involving monomials of critical and non-critical parameters.

#### Eliminating slow species: averaging

Averaging is an useful model reduction technique for high-dimensional clocks or for other types of oscillating molecular systems (the activity of some transcription factors, among which NF-*κ*B, present oscillations under some conditions).

Averaging can be applied rather generally [22-24] to produce coarse grained quantities and reduced models. The typical mathematical result applying here is due to Pontryagin and Rodygin [48,49]. Supposing that the oscillating species are *x*, the non-oscillating species are *y*, and ∈ is a small parameter, then we have:

(30) $$\epsilon \frac{dx}{dt}=f(x,y)$$

(31) $$\frac{dy}{dt}=g(x,y)$$

It is supposed that for any *y*, the fast dynamics (30) has an attractive hyperbolic limit cycle *x *= *ψ *(*τ*, *y*), of period *T*(*y*): *ψ *(*τ *+ *T*(*y*), *y*) = *ψ*(*τ*, *y*) (*τ *= *t*/*ϵ*). Then, after a short transient, the slow variable satisfies the averaged equation:

(32) $$\frac{dy}{dt}=\frac{1}{T(y)}\int_{0}^{T(y)}g(\psi (\tau ,y),y)d\tau$$

The result can be extended to the case when *x *= *ψ*(*τ*, *y*) describes damped oscillations, with damping time much larger than *ϵ*, i.e. when the fast dynamics (30) has a stable focus and the eigenvalues of the Jacobian ∂*f*/∂*x *calculated at the focus are of the form -*λ *± *iμ*, 0 <*λ *<<*μ *= O(1/*ϵ*).

The following averaged steady state equation allows to eliminate the slow species *y*:

(33) $$\int_{0}^{T(y)}g(\psi (\tau ,y),y)d\tau =0$$

If (32) has a stable steady state, we always reach this situation. In this case, the slow non-oscillating variables *y *are constant in time and can be considered to be conserved, which has two significant consequences.

First, Eq.(33) restores conservation. Slow variables are often the result of broken conservation laws. In fact, in biological open systems, nothing is conserved. Conservation laws result from balancing production and degradation either passively (slow processes) or actively (feed-back). Thus, we can ignore production and degradation of molecules whose level is rigorously controlled. Eq.(33) describes such a case.

Second, (33) are averaged steady state equations for the slow variables. If slow variables *y *reach stationarity, the only variables that change in time are the oscillating variables *x*. Eq.(30) describes the dynamics of *x*, considering that *y *satisfies (33). For oscillators, averaging provides a way to eliminate slow non-oscillating variables, which is formally equivalent to quasi-stationarity and represents a new case of applicability of Clarke's method. The difference between the two cases is that we eliminate fast variables by solving quasi-stationarity equations and we eliminate slow variables by solving averaged stationarity equations. Thus, intermediate non-oscillating variables are expressed in terms of only non-oscillating terminal variables. If there are no non-oscillating terminal variables, then non-oscillating intermediates become conserved quantities.

## Results and discussion

### Methodology

In this section, we demonstrate hierarchical model reduction, model comparison and critical parameter identification. Critical parameters are identified during the reduction procedure.

Model reduction starts with a complex model, from which we obtain a hierarchy of reduced models by eliminating various intermediate species. The intermediate species are either quasi-stationary species (in general), or non-oscillating species (for oscillators). The complexity of a model is quantified by three integers. A model with *n *species, *r *reactions and *p *parameters is designated by *M*(*n, r, p*). Our conception about systems biology models is summarized by the following idea. Instead of providing a single model, it is better to provide a hierarchy of models, and the relations between them. Depending on the application, we can choose the most appropriate model in the hierarchy or couple several simple models into a larger model.

The number of parameters in a model are obtained as follows. If the elementary reactions follow mass action law kinetics, there are *n*_*k *_= 2*n*_*r *_+ *n*_*i *_kinetic constants, where *n*_*r*_, *n*_*i *_are the numbers of reversible and irreversible reactions. Reactions with kinetic constants zero are not considered in the counting. Each one of the *n*_*c *_conservation laws adds an extra parameter, the value of the conserved quantity. These values follow from initial data and are important parameters for the dynamics. For multi-compartment models, the ratios of the compartments volumes (in the example below there is only one ratio *kv*, the cytoplasm to nucleus volume ratio) are extra parameters. Thus *p *= *n*_*k *_+ *n*_*c *_+ 1.

Model comparison has a similar flowchart. By model comparison we understand a) mapping one model to another one by model reduction or mapping each model to a third one, closest in some sense to both; b) compare predictions of the models (for instance, about how the system responds to perturbations) for sets of parameters related one to the other by the mapping at a). In this case, the choice of intermediate species is dictated by the differences between the models to be compared.

### Hierarchy of models for NF-*κ*B signalling

The transcription factor NF-*κ*B is involved in a wide diversity of domains such as the immune and inflammatory responses, cell survival and apoptosis, cellular stress and neuro-degenerative diseases, cancer and development. NF-*κ*B is sequestered in the cytoplasm by inactivating proteins named I*κ*B. Upon signalling, I*κ*B molecules are phosphorylated by a kinase complex, then ubiquitinylated, and finally degraded by the proteasomal complex. NF-*κ*B bound to I*κ*B molecules is then transported to the nucleus to activate its target genes. There are known five members of the NF-*κ*B family in mammals, Rel (c-rel), RelA (p65), RelB, NF-*κ*B1 (p50 and its precursor p105) and NF-*κ*B2 (p52 and its precursor p100). This generates a large combinatorial complexity of dimers, affinities and transcriptional capabilities. I*κ*B family comprises seven members in mammals (I*κ*B*α*, I*κ*B*β*, I*κ*B*ϵ*, I*κ*B*γ*, Bcl-3) [50]. All these inhibitors display different affinities for NF-*κ*B dimers, multiplying the combinatorial complexity. Moreover, the gene coding for I*κ*B*α*, is transcriptionally activated by NF-*κ*B. This negative feed-back loop can give rise to oscillations of the activity of NF-*κ*B [51,52]. Phosphorylation of I*κ*B*α *upon signalling is provided by a kinases complex that includes IKK*α *and IKK*β *(I*κ*B Kinase, also named IKK1 and IKK2), associated to a regulating protein NEMO (NF-*κ*B Essential Modulator, also called IKK*γ*). Therefore, it is clear that understanding such a complex biological system requires modeling. Several mathematical models of NF-*κ*B have been published. The first model described a single NF-*κ*B molecule, which binds to I*κ*B*α*, I*κ*B*β *and I*κ*B*ϵ*. This work demonstrated oscillations in NF-*κ*B activity, confirmed by experimental data [51]. The model set by [53] included in addition an A20 molecule whose production is enhanced upon NF-*κ*B stimulation, and which negatively regulates IKK activity. A third model analyzed the critical parameters necessary for maintaining oscillations, with given amplitude and frequency [54]. In addition, a minimal simplified model was also set to study the oscillations of the NF-*κ*B module [55]. We propose here a fourth, new model, with more complex descriptions that takes into account transcription, translation and degradation of different NF-*κ*B units.

In our model, NF-*κ*B is considered to be made of two subunits, p50 and p65. All combinations of these subunits are allowed, including two homodimers p50:p50, p65:p65 and one heterodimer p50:p65. The three dimers of NF-*κ*B are characterized by different affinities for DNA sites, and associate differentially to I*κ*B*α*, *β *and *ϵ*, generating thus 9 species with different abundances and characteristics upon signalling and degradation. The production of the dimer p65 is considered under control of a transcription factor FTAy, which represents a simplification of many transcription factors supposed to activate this promoter. p50 is produced from a precursor molecule p105. The transcription factor FTAx binds to the promoter of p105 to activate its transcription at a basal level. Similarly to FTAy, this factor represents the sum of individual activities due to several transcription factors contributing to the basal activity of this promoter. As the p105 gene is activated by NF-*κ*B, this factor can also bind to the p105 promoter and activate the transcription above the basal level. Promoter of I*κ*B is controlled by NF-*κ*B and FTAz in a similar way as it is p105. In addition, it was supposed that nuclear I*κ*B can come and bind to NF-*κ*B when this is on the promoter of I*κ*B or of p105. Once the complex formed this can unbind from the DNA, taking NF-*κ*B away. The kinase activation/inactivation module including interactions with A20 was borrowed from [53]. Let us notice that transcription regulation modules are very simplified and do not take into account specificities of eukaryotic regulation (existence of several binding sites, enhancers, etc).

Initiation [56,57] and elongation [58-60] for transcription and translation rates come from previous studies which were more recently re-examined [61]. Binding and unbinding constants for NF-*κ*B subunits come either from literature [62-64] or from previous models [51,53].

We should signal large uncertainty concerning values of constants. For example, the rate of degradation of I*κ*B was assumed to be independent of the state of the molecule, either free or bound to NF-*κ*B. This led to a poor fit of computational simulations of the NF-*κ*B signalling module. The rate was newly measured in vivo and led to better fits of I*κ*B levels and basal NF-*κ*B activity [65]. This motivated us to determine which parameters of the model are critical and should be known with precision and which ones are not critical.

A simplified version of the model (considering only the I*κ*B*α *inhibitor) is given in Table 1.

**Table 1:** Model ℳ(39, 65, 90).

reaction	kinetic constants
IKK → IKK|active	k1 = 0.0025
IKK → null	k2 = 0.000125
null → IKK	k3 = 1e-005
IKK|active + A20 → A20 + IKK|inactive	k4 = 0.1
IKK|active → IKK|inactive	k5 = 0.0015
IKK|active → null	k6 = 0.000125
IKK|active + IkBa@csl → IKK|active:IkBa	k7 = 0.24
IKK|active:IkBa → IKK|active	k8 = 0.1
IKK|active + IkBa:p50:p65@csl → IKK|active:IkBa:p50:p65	k9 = 1.2
IKK|active:IkBa:p50:p65 → IKK|active + p50:p65@csl	k10 = 0.1
IKK|inactive → null	k11 = 0.000125
IkBa:p50:p65@csl → p50:p65@csl	k12 = 2e-005
p50:p65@csl + IkBa@csl ⇌ IkBa:p50:p65@csl	kf13 = 0.5 kr13 = 0
p50:p65@ncl + IkBn ⇌ IkBa:p50:p65@ncl	kf14 = 0.5 kr14 = 0
p50:p65@csl ⇌ *k*_*v *_p50:p65@ncl	kf15 = 0.0025 kr15 = 8e-005
mRNAA20 → mRNAA20 + A20	k16 = 0.5
mRNAA20 → null	k17 = 0.0004
A20 → null	k18 = 0.0003
null → mRNAA20	k19 = 0
p50:p65@ncl → p50:p65@ncl + mRNAA20	k20 = 5e-007
IkBa@csl → null	k21 = 0.0001
mRNAIkBa → mRNAIkBa + IkBa@csl	k22 = 0.5
IkBa@csl ⇌ *k*_*v *_IkBn	kf23 = 0.001 kr23 = 0.0005
null → mRNAIkBa	k25 = 0
mRNAIkBa → null	k27 = 0.0004
*k*_*v *_IkBa:p50:p65@ncl → IkBa:p50:p65@csl	kf28 = 0.01 kr28 = 0

Prop105:RNAP + FTAx ⇌ Prop105:RNAP:FTAx	kf32 = 10 kr32 = 0.0001
Prop105:RNAP → Prop105:RNAP + RNAP1|active	k33 = 0.0005
Prop105:RNAP:FTAx → Prop105:RNAP:FTAx + RNAP1|active	k34 = 0.1
RNAP1|active → mRNAp105	k35 = 0.01
mRNAp105 → mRNAp105 + p105	k36 = 0.0041
mRNAp105 → null	k37 = 5e-005
p105 → null	k38 = 6e-005
p105 → p50	k39 = 0.00013
p50 → null	k40 = 6.4e-005
FTAy + Prop65:RNAP ⇌ Prop65:RNAP:FTAy	kf41 = 10 kr41 = 0.0001
Prop65:RNAP → Prop65:RNAP + RNAP2|active	k42 = 0.0005
Prop65:RNAP:FTAy → Prop65:RNAP:FTAy + RNAP2|active	k43 = 0.1
RNAP2|active → mRNAp65	k44 = 0.016
mRNAp65 → mRNAp65 + p65	k45 = 0.0053
mRNAp65 → null	k46 = 5e-005
p65 → null	k47 = 6.4e-005
FTAz + ProIkBa:RNAP ⇌ ProIkBa:RNAP:FTAz	kf48 = 10 kr48 = 0.0001
ProIkBa:RNAP → ProIkBa:RNAP + RNAP3|active	k49 = 0.0005
ProIkBa:RNAP:FTAz → ProIkBa:RNAP:FTAz + RNAP3|active	k50 = 0.02
RNAP3|active → mRNAIkBa	k51 = 0.025
p50 + p65 ⇌ p50:p65@csl	kf52 = 0.003 kr52 = 0.001
p50:p65@csl → null	k53 = 0.0002
p50:p65@ncl → null	k54 = 0.0002
p50:p65@ncl + ProIkBa:RNAP ⇌ ProIkBa:RNAP:p50:p65	kf55 = 0.62 kr55 = 0.00048
p50:p65@ncl + ProIkBa:RNAP:FTAz ⇌ ProIkBa:RNAP:FTAz:p50:p65	kf56 = 0.62 kr56 = 0.00048
IkBn + p50:p65@nclProIkBa:RNAP ⇌ ProIkBa:RNAP:p50:p65:IkBa	kf57 = 18.4 kr57 = 0.055
IkBn + ProIkBa:RNAP:FTAz:p50:p65 ⇌ IkBnProIkBa:RNAP:FTAz:p50:p65	kf58 = 18.4 kr58 = 0.055
ProIkBa:RNAP:p50:p65:IkBa ⇌ IkBa:p50:p65@ncl + ProIkBa:RNAP	kf59 = 0.0038 kr59 = 8e-013
IkBnProIkBa:RNAP:FTAz:p50:p65 ⇌ IkBa:p50:p65@ncl + ProIkBa:RNAP:FTAz	kf60 = 0.0038 kr60 = 8e-013
p50:p65@nclProIkBa:RNAP → p50:p65@nclProIkBa:RNAP + RNAP3|active	k61 = 0.06
ProIkBa:RNAP:FTAz:p50:p65 → ProIkBa:RNAP:FTAz:p50:p65 + RNAP3|active	k62 = 0.6
p50:p65@ncl + Prop105:RNAP ⇌ Prop105:RNAP:p50:p65	kf63 = 0.62 kr63 = 0.00048
p50:p65@ncl + Prop105:RNAP:FTAx ⇌ Prop105:RNAP:FTAx:p50:p65	kf64 = 0.62 kr64 = 0.00048
IkBn + Prop105:RNAP:p50:p65 ⇌ Prop105:RNAP:p50:p65:IkBa	kf65 = 18.4 kr65 = 0.055
IkBn + Prop105:RNAP:FTAx:p50:p65 ⇌ Prop105:RNAP:FTAx:p50:p65:IkBa	kf66 = 18.4 kr66 = 0.055
Prop105:RNAP:p50:p65:IkBa ⇌ IkBa:p50:p65@ncl + Prop105:RNAP	kf67 = 0.0038 kr67 = 8e-013
Prop105:RNAP:FTAx:p50:p65:IkBa ⇌ IkBa:p50:p65@ncl + Prop105:RNAP:FTAx	kf68 = 0.0038 kr68 = 8e-013
Prop105:RNAP:p50:p65 → Prop105:RNAP:p50:p65 + RNAP1|active	k69 = 0.006
Prop105:RNAP:FTAx:p50:p65 → Prop105:RNAP:FTAx:p50:p65 + RNAP1|active	k70 = 0.06
IkBa:p50:p65@csl → null	k71 = 0.0002
IkBa:p50:p65@ncl → null	k72 = 0.0002

Detailed description of the complex model for NF-κB signalling. The names of the species are provided following the template similar to that proposed in B7676: Entity1name|Modifications ...: Entity2name|Modifications...[|active]@compartment. Here, the colon symbol ':' delimitates components of a complex. Optional suffix 'active' describes the active state of the protein. The localization information (@compartment suffix) is provided when a protein or complex exists in both nucleus (@ncl) and cytoplasm (@csl). Mass action law constants are either in s-1 or in μMs-1 units. kv parameter (cytoplasm/nucleus volume ratio) is set up to 5. First reactions in the list (Re1–Re28) correspond to the Lipniacky's model.

### Model reduction

As an illustration of the model reduction flowchart, we obtain from the model proposed by Lipniacki [53] a series of simpler models. This model is ℳ(14, 25, 28) in our hierarchy: it contains only one reversible reaction and the total NF-*κ*B quantity is conserved *n*_*c *_= *n*_*r *_= 1. The description of the reactions can be found in Table 1 (Lipniacki's model is a submodel of our model).

The model was forced to function in a strongly oscillating regime. This situation is the most unfavorable for the simple version of Clarke's method which is doomed to shorten delays and to destabilize oscillations when intermediates are not appropriately chosen. Thus, it represents a good test for our method. First, we identify quasi-stationary and non-oscillating species. We define log-average concentration *c*_log _= log <*c *> and the log-amplitude *a*_log _= min(log max(*c*) - *c*_log_, *c*_log _- log min(*c*)) (the minimum is to avoid divergence when min(*c*) = 0). Species whose log-amplitudes are low and well separated from other values are declared non-oscillating. In order to detect quasi-stationary species, for each species *A*_*i *_we compare two trajectories (concentrations as functions of time): a) the trajectory in the unreduced model b) the trajectory of *A*_*i *_calculated from the trajectories of the species influencing *A*_*i *_by using the quasi-stationarity equation (22) for *I *= {*i*}. The two trajectories must be close one to another for quasi-stationary species (except for a short transition region), see Fig. 3a). Hausdorff distance between the two trajectories can be used to detect quasi-stationary species for automatic computation. Non-oscillating species could also satisfy this criterion, but after a larger transition region, because they are slow (see the behavior of IKK|inactive in Fig. 3a)).

These procedures allow to identify 7 quasi-stationary species (IKK|active, IKK, IKK|active:IkBa, IKK|active:IkBa:p50:p65, IkBa@ncl, IkBa:p50:p65@ncl, p50:p65@csl) and one non-oscillating species (IKK|inactive). Two species with small concentration (mRNAA20, mRNAIkBa) are not quasi-stationary, as their relaxation time can be compared to the period of the oscillations. The smallness of their concentration is not a consequence of rapid consumption, but of small production (transcription) rate. Two species with large concentration are quasi-stationary (IkBa@ncl, p50:p65@csl).

The 8 intermediate species can be grouped into two connected subsets (modules). The first module involves six cytosol located intermediates (IKK|active, IKK|inactive, IKK, IKK|active: IkBa, IKK|active:IkBa:p50:p65, p50:p65@csl) and four terminal species (A20, IkBa@csl, IkBa:p50:p65@csl, p50:p65@ncl). The intermediate reactions form the cytoplasmic part of the signalling mechanism. The kinase transformation reactions *R*_1–11_, the complex release reaction *R*_12_, the complex formation reaction *R*_13 _and the NF-*κ*B translocation reaction *R*_15 _are replaced by two simple sub-mechanisms representing the modulated inhibitor degradation (IkBa → ∅), and summarizing the NF-*κ*B release and translocation (IkBa:p50:p65@csl → p50:p65@ncl), respectively. The corresponding dominant rates are:

(34) $${R^{\prime }}_{21}\approx k_{21p1}x_{10}x_{13}/((k_{21p2}+x_{10})(k_{21p3}+x_{8}))$$

(35) $${R^{\prime }}_{15}\approx k_{15p1}x_{13}/((k_{15p2}+x_{10})(k_{15p3}+x_{8}))$$

where *x*_10 _= [*IkBa*@*csl*], *x*_8 _= [*A*20], *x*_13 _= [*IkBa *: *p*50 : *p*65@*csl*], *k*_21*p*1 _= *k*_3_*k*_9_/*k*_4_, *k*_21_*p*_2 _= *k*_15_*p*_2 _= *k*_15_/*k*_13_, *k*_15_*p*_2 _= (*k*_15_*k*_3_*k*_9_)/(*k*_4_*k*_13_), *k*_21_*p*_3 _= *k*_15_*p*_3 _= *k*_5_/*k*_4_.

After reduction of the first module we obtain the model ℳ(8, 12, 19).

The second module is situated in the nucleus and contains IkBa@ncl and IKBnp50:p65@ncl. Three intermediate reactions (translocations of inhibitor and of the complex and complex formation) are replaced by one simple submechanism describing the nuclear complex formation and translocation (IkBa@csl + p50:p65@ncl → IkBa:p50:p65@csl) whose dominant rate is:

(36) $${R^{\prime }}_{14}=k_{14p1}x_{10}x_{7}/(k_{14p2}+x_{7})$$

where *x*_7 _= [*p*50 : *p*65@*ncl*], *k*_14*p*1 _= *k*_23_, $k_{14p2}=k_{v}{k^{\prime }}_{23}/k_{14}$.

This reduction step leads to the model ℳ(6, 10, 17). The dynamics (illustrated by trajectories in Fig. 3b) of the two new models is practically the same as the dynamics of the non-reduced model. One should not expect a perfect match because the method is based on asymptotic order relations between parameters. In establishing the expression of dominant rates we have considered that one parameter is much bigger than another one if the absolute value of their ratio is larger than ten. Of course, a more drastic criterion would produce more complex expressions, because less monomials could be simplified (separation of these monomials would not be large enough).

We have tested reduction of two more species that have small concentration but are not quasi-stationary. Reducing the species mRNAA20 leads to the model ℳ(5, 8, 15) Intermediate reactions (representing the transcription/translation module) are replaced by a single one (production of protein), of parameter *k*_20*p *_= *k*_16_*k*_20_/*k*_17_. This model has stable oscillations, but with slightly smaller period, and with different phase relations between oscillating species (A20 is almost in phase with nuclear NF*κ*B). Both period and phase changes result from the reduced delay on the negative feed-back loop containing A20. Reducing the species mRNAIkB has destabilizing effect on the oscillations. It is no longer possible to obtain self-sustained oscillations and damping times are generally smaller than for the non-reduced model. It is well known that delayed negative feed-back favors stable oscillations and that reducing the delay destabilizes oscillations. Our findings suggest that the delay along the IkBa negative feed-back loop is more important for the stability of the oscillations than the delay along the A20 loop.

Model reduction allows to identify critical and non-critical parameters. Parameters of reduced models are monomials of parameters of the non-reduced models (see Eqs.(34),(35),(36)). Some parameters of the non-reduced model may not occur in these monomials; these are non-critical parameters. Among monomials, only some are critical. Critical monomials are detected by sensitivity studies [66] performed on the reduced model. Critical parameters of the non-reduced model are contained in the critical monomials of the reduced model. The relation between critical parameters and critical monomials is hierarchical: monomials may be combined to form new monomials (in our example only two hierarchical levels are present). The degrees of critical monomials provide qualitative information on the influence of various critical parameters on the properties of the system. For instance, if two parameters have degrees of opposite signs in a critical monomial, their effects will be opposite.

**Figure 4:**
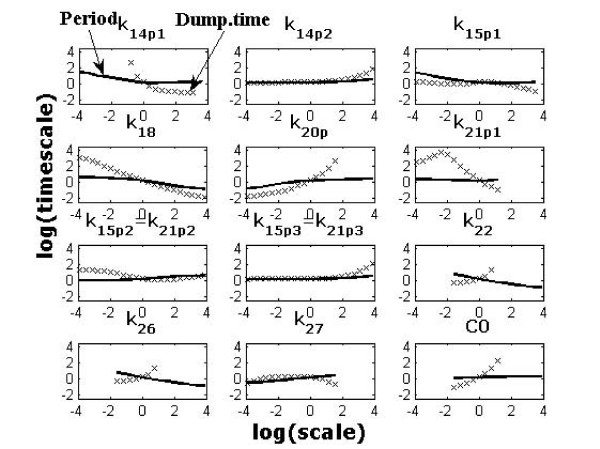
**Log-log sensitivity of the damping time and of the period of the oscillations with respect to variations of different parameters of the model**ℳ**(5, 8, 15)**. The parameters are multiplied by a scale *s *∈ (1/50, 50). The log(timescales) are represented as functions of log(*s*). Period and damping time are not represented on intervals of parameter values where oscillations are over-damped (the ratio of the damping time to the period is smaller than 1.75). Damping time is infinite and not represented for intervals of parameter values where oscillations are self-sustained. The latter intervals are limited by Hopf bifurcations where the damping time diverges.

**Figure 5:**
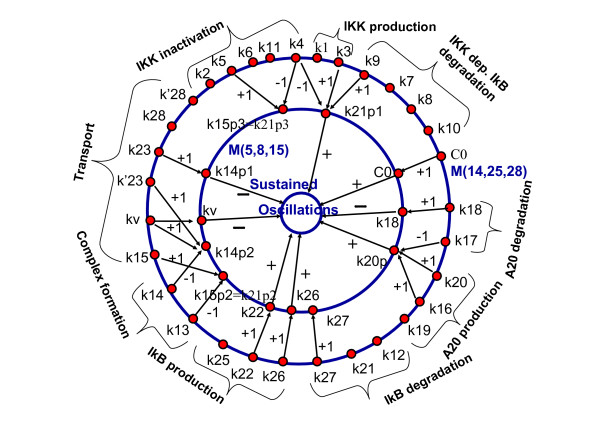
**Correspondence between the parameters of the models**ℳ**(14, 25, 28)** and ℳ**(5, 8, 15)**. Parameters of the first model are gathered into monomials that are parameters of the reduced model. The integers on the arrows connecting parameters represent the corresponding powers of the parameters in the monomial. The critical monomials are connected to the property on which they act upon (here sustained oscillations). Thus, an increase of *k*_21*p*1 _= *k*_2_*k*_9_$k_{4}^{-1}$ favors significantly the oscillations.

As an example, we detect critical monomials in the simplest reduced model ℳ(5, 8, 15), first with respect to damping time and then with respect to the period of the oscillations. Deciding rigorously what large sensitivity means is not easy. In [34] we proposed a criterion which applies to properties that are homogeneous of degree ±1 in the kinetic constants, in particular, to characteristic times. Let *τ *be the studied quantity and *k *the parameter (monomial). We say that *k *is critical if $\sup_{|\log(s)|<A}|\frac{d\log\tau (sk_{0})}{d\log s}|>1$, where *A *> 0 is some fixed constant and *k*_0 _some central value of the parameter. The sensitivity study is presented in Fig. 4. The relation between parameters of the initial and the reduced models is represented in Fig. 5. Damping time of the oscillations is most sensitive to parameters *k*_14*p*1_, *k*_18_, *k*_20*p*_, *k*_21*p*1_, *k*_22_, *k*_26_, *C*_0_. By changing these parameters, the oscillations can be modified from damped to self-sustained. The above parameters are the critical monomials from which we get the critical parameters (with respect to damping time) of the unreduced model: *k*_23_, *k*_18_, *k*_16_, *k*_20_, *k*_17_, *k*_3_, *k*_9_, *k*_4_, *k*_22_, *k*_26_, *C*_0_. The degrees of the critical monomials represent logarithmic sensitivities, therefore they provide both sign an strength of the influence of the critical parameters on the studied property. For instance, from *k*_21*p*1 _= *k*_3_*k*_9_(*k*_4_)^-1 ^we can say that damping time can be increased (produce sustained oscillations) by reducing *k*_3_, or by reducing *k*_9_, or by increasing *k*_4_), see also Fig. 3.

Critical parameters correspond to reactions affecting three targets: the kinase, A20, and the inhibitor, see Fig. 5. Four groups of critical monomials are easy to interpret. Increase of the monomial *k*_20*p *_stands for increasing the NF-*κ*B dependent A20 production (changing *k*_17_, *k*_18 _have the opposite effect, increase degradation). Increasing *k*_26_, *k*_22 _stands for increasing the NF-*κ*B dependent I*κ*B production. The latter effect has been exploited in [52] to stabilize oscillations by transfecting HeLa cells with *κ*I*κ*B-EGFP vector. Decreasing *k*_14*p*1 _stands for decreasing the nuclear concentration of the inhibitor, by reducing its translocation rate to the nucleus. It is possible that the experiment in [52] affected also this constant (in the right direction, ie towards decreased translocation rate) by attaching EGFP to the inhibitor. The critical monomial *k*_21*p*1 _is more difficult to interpret in terms of putative targets. It gathers recovery (via *k*_3_) and dynamical properties (via *k*_9_), as well as the A20 dependent inactivation (via *k*_4_) of the kinase IKK. Finally, increasing *C*_0 _means increasing the total concentration of NF-*κ*B (free or trapped).

The value of the period is remarkably robust. There are no critical monomials for the period.

Although the strongest effect on the oscillations has already been tested experimentally by increasing the NF-*κ*B dependent I*κ*B production [52], there are two remaining targets (the kinase and A20) that could be tested experimentally.

The sequence of reduction steps described above is illustrated on Fig. 1S in Additional File 1. A series of simplified models provided in SBML 2.1 [67] format and annotated by CellDesigner 3.5 [68] software are submitted to BioModels database  with the following ids: MODEL7743386835, MODEL7743358405, MODEL7743315447, MODEL7743212613.

### Model comparison

To illustrate model comparison, we compare a version of our complex model (that employs only the most important member of the I*κ*B family, namely I*κ*B*α*) to the model ℳ(14, 25, 28), proposed by Lipniacki [53]. Our model is ℳ(39, 65, 90 (there are 39 species, 65 reactions, among which 18 are reversible, 6 conservation laws, though the total NF-*κ*B quantity is not conserved). The model ℳ(14, 25, 28) is a submodel of ℳ(39,65, 90) in the sense that all its species are included in our larger model. The description of our model has been sketched at the beginning of the section 3.2. A complete description is given in Table 1. To perform model comparison we define the set of intermediates *I *as the difference of the sets of species of the two models. There are 25 intermediate species and a small frontier: only 5 terminal species.

In order to verify that intermediates can be eliminated with no consequence on the dynamics we have used the method described in the previous section.

**Figure 6:**
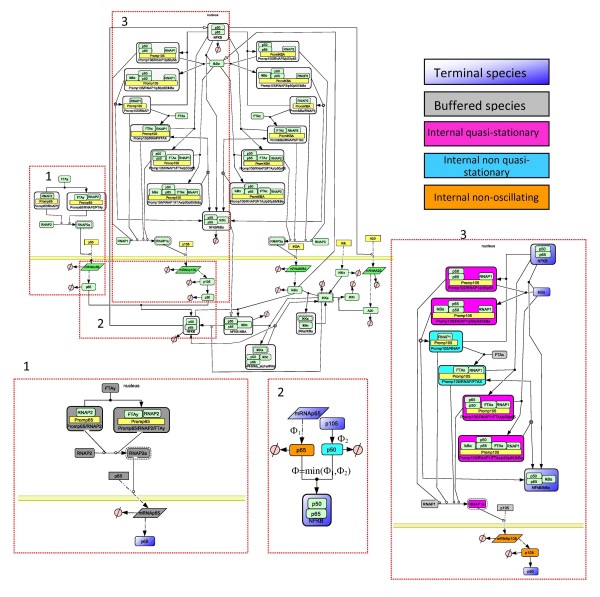
**Complete model **ℳ**(39, 65, 90) (left, top)**. Intermediate mechanisms for 1) Production module of p65; 2) Min-funnel production of p50:p65@csl; 3) Production module of p50.

**Figure 7:**
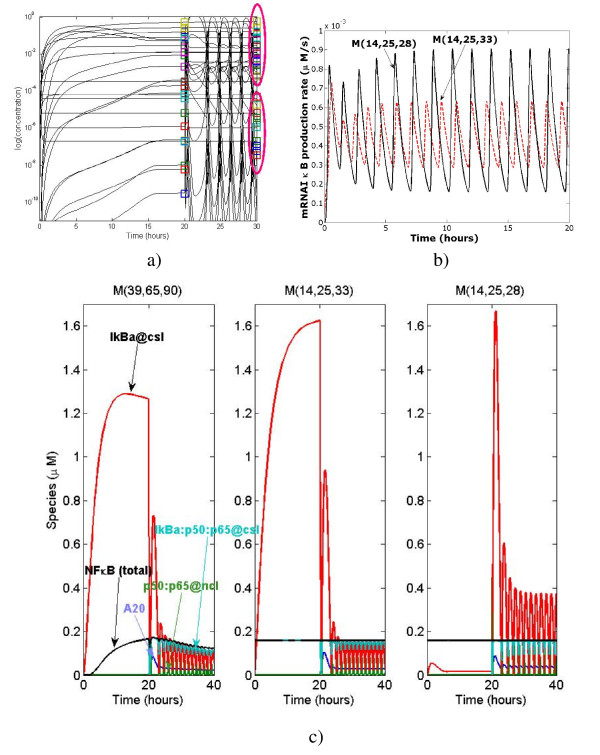
**Model comparison a) Trajectories of various species for the model *M *(39, 65, 90); quasi-stationary species have concentrations in the lower cluster.** b) Production rates of mRNAI*κ*B for two models having the same reactions and species, differing only by one kinetic law. c) Trajectories (signal applied at t = 20). Notice the different behavior of *IkBa*@*csl *in ℳ(14, 25, 28).

The intermediate species can be divided into four functional modules: production of mRNAp50, production of mRNAp65, production of mRNAI*κ*B, and min funnel production of the complex p50:p65@csl, see Fig 6. We found three categories of intermediates. There are 10 quasi-stationary species, 3 non-oscillating species and 7 buffered species (species in large excess whose concentrations are practically constant). The elimination of these is entirely justified and has no consequence on the oscillations. There are 5 non-quasistationary, oscillating species. Among these, 4 are low concentration species, representing the states of two promoters (Prop105:RNAP, PropIkBa:RNAP) free and singly occupied by transcription factors FTAx, FTAz, respectively. However, we can safely eliminate them because transcription initiation starts dominantly when both p50:p65@csl and FTAx (or FTAz) are on the promoter, therefore the non-quasistationary promoter states are not important. The last non-quasistationary, oscillating species is p50 who binds to p65 (another slow, but non-oscillating species) to produce p50:p65@csl via the min funnel. Concentrations of all quasi-stationary intermediates are small (see Fig. 7a)), (< 10^-4 ^*μM *corresponding to less than 30 molecules per cell). The reduction that we propose is fully justified for a deterministic model, but one may ask if deterministic differential equations apply in this case. We have shown elsewhere [33] that deterministic approximation can be applied in two different situations. The first, well known situation is when the numbers of molecules are large; the law of large numbers applies. The second, less known situation, is when some species are in small numbers, but when the reactions involving these species are frequent. An example is the quick binding-unbinding of a transcription factor on a promoter site. In this case, we can consider that various states of the promoter are at stochastic equilibrium (meaning they have reached a time invariant probability distribution). Under some conditions (the intermediate reactions should be pseudo-monomolecular), stochastic averaging [69] of the remaining equations (describing the promoter activity) with respect to the invariant distribution is equivalent to applying quasi-stationarity to the fast concentrations in the deterministic approach.

Reduction can be decomposed into several steps. The first three steps correspond to simplifications of the mechanisms producing the proteins *p*_50_, *p*_65_, and the mRNAIkBa. Thus, the reactions *R*_41–46_, *R*_32–39_, *R*_63–70_, *R*_48–51_, *R*_55–62 _are replaced by the simple submechanisms ∅ → p_65_, ∅ → p_50_, ∅ → mRNAIkBa, of rates ${R^{\prime }}_{45},{R^{\prime }}_{39},{R^{\prime }}_{26}$, respectively. The quasi-stationarity equations become linear after applying the strong binding, large concentration approximation for the transcription factors FTAx-y-z. The corresponding linear mechanisms $R_{I}$ are represented in Fig. 6. The dominant solutions of the quasistationarity equations are obtained with techniques presented for linear subsystems. Using also Eq.(28) we find the following simple submechanism rates:

(37) $${R^{\prime }}_{45}\approx \frac{k_{43}k_{45}}{k_{46}}{\left[Prop65:RNAP\right]}_{0},$$

(38) $${R^{\prime }}_{39}\approx \frac{k_{39p1}x_{11}+k_{39p2}x_{7}}{x_{7}x_{11}+k_{39p3}x_{11}+k_{39p4}x_{7}}$$

(39) $${R^{\prime }}_{26}\approx \frac{k_{26p1}x_{11}+k_{26p2}x_{7}}{x_{7}x_{11}+k_{26p3}x_{11}+k_{26p4}x_{7}}$$

Where *x*_7 _= [p50 : p65@*ncl*], *x*_11 _= [*IkBa*@*ncl*], $k_{39p1}=\frac{k_{39}}{k_{38}+k_{39}}\frac{k_{36}k_{34}k_{68}P_{0}^{105}}{k_{37}k_{64}}$, $k_{39p2}=\frac{k_{39}k_{36}}{k_{38}+k_{39}}\frac{{k^{\prime }}_{66}k_{70}P_{0}^{105}}{k_{37}k_{66}}$, $k_{39p3}=\frac{k_{68}}{k_{64}}$, $k_{39p4}=\frac{{k^{\prime }}_{66}}{k_{66}}$, $k_{26p1}=\frac{k_{50}k_{60}P_{0}^{IkBa}}{k_{56}}$, $k_{26p2}=\frac{{k^{\prime }}_{58}k_{62}P_{0}^{IkBa}}{k_{58}}$, $k_{26p3}=\frac{k_{60}}{k_{56}}$, $k_{26p4}=\frac{{k^{\prime }}_{58}}{k_{58}}$. $P_{0}^{105}$, $P_{0}^{IkBa}$ are the concentrations of promoter sites of p105, IkBa, respectively.

The fourth step is a min funnel simplification of the production of the complex *p*50 : *p*65@*csl*.

${R^{\prime }}_{39}$, *R*_40_, ${R^{\prime }}_{45}$, *R*_47_, *R*_52 _are replaced by ∅ → p50:p65@csl, of rate:

(40) $${R^{\prime }}_{52}\approx min({R^{\prime }}_{39},{R^{\prime }}_{45})={R^{\prime }}_{39}$$

This leads to the model ℳ(14, 30, 41).

The fifth step, justified by averaging, introduces a new conservation law (the model ℳ(14, 30, 41) has no conservation law). Without the reactions ${R^{\prime }}_{52}$, *R*_53–54_, *R*_71–72_, that produce and consume *p*50 : *p*65, the total amount of *p*50 : *p*65 (free or in complexes with other species) would be conserved. Considering that the degradation reactions *R*_53–54_, *R*_71–72_, *R*_72 _have the same constant *k*, the total amount of NF-*κ*B (free, or in complexes) represented by the variable

*C *= [*p*50 : *p*65@*csl*] + [*p*50 : *p*65@*ncl*]/*k*_*v *_+ [*p*50 : *p*65 : *IkBa*@*csl*] + ... satisfies the equation :

(41) $$\frac{dC}{dt}=-kC+{R^{\prime }}_{52}$$

The dynamics (41) has two time scales, a slow timescale 1/*k *and a rapid timescale, the period of the oscillations (${R^{\prime }}_{52}$ is oscillating). *C *is a slow, non-oscillating variable, it averages oscillations. Thus, the asymptotic, total amount of NF-*κ*B is:

(42) $$C_{0}=<{R^{\prime }}_{52}>/k,$$

where the average is over a period of the oscillations.

In the fifth reduction step, reactions *R*_52_, *R*_53_, *R*_54_, *R*_71_, *R*_72 _are eliminated. Initial conditions of the system are chosen such that initial total NF-*κ*B is *C*_0 _(this is a conserved value and a new parameter of the model).

We obtain the model ℳ(14, 25, 33) that has the same species and reactions as Lipniacki's model ℳ(14, 25, 28), but slightly more parameters. The difference in the number of parameters comes from the more complex expressions of the mRNA I*κ*B transcription rate ${R^{\prime }}_{26}$ given by (39). In our model this rate is modulated by the nuclear I*κ*B concentration *x*_11 _(indeed, the inhibitor can unbind NF-*κ*B from DNA). This phenomenon is not taken into account in [53] where the corresponding rate is simply *R*_26 _= *k*_26_*x*_7_.

One important objective of model comparison is to obtain the parameter mapping. This allows to calculate the parameters of one model if the parameters of the other model are known. Then, dynamical properties of the models can be compared. In our example, all the parameters of ℳ(14, 25, 28) should be equal to the corresponding parameters of ℳ(39, 65, 90) except for *C*_0 _and *k*_26 _which are obtained by averaging (*C*_0 _is given by Eq.(42) and *k*_26 _is calculated in order to have equal average production rates of mRNAI*κ*B in the two models, see Fig. 7b). Dynamical comparison has been done in Fig. 7c. The model ℳ(14, 25, 33) is a reduction of ℳ(39, 65, 90), therefore the dynamics of these two models is very similar. The model ℳ(14, 25, 28) preserves the main features of the dynamics, except for the behavior I*κ*Ba. Without signal, the quantity of inhibitor in ℳ(14, 25, 28) is small (it is largely in excess in the other two models). With signal, the amplitude of the oscillations is higher in ℳ(14, 25, 28). These differences follow from the different kinetic laws for the transcription of I*κ*Ba. Basically, in *M*(14, 25, 33) and ℳ(39, 65, 90), I*κ*Ba has some negative influence on its own production (see Eq.(39)).

Our most complex model can account for phenomena that can not be studied by any of the conservative models ℳ(14, 25, 28), ℳ(14, 25, 33), namely it can take into account variations of the NF-*κ*B total quantity. Although this is not important in normal situations (when *C *is conserved), it could become important if one wants to cope with strong perturbations of NF-*κ*B activity.

Thus, using a more complex model depends on the experimental situation (number of variables that can be observed, or controlled, type of perturbation). The role of mathematics and modeling in quantitative biology is to predict the behavior of a system. Depending on which behavior, the simplest theory can change, and we want a hierarchy of models and model mapping methods. The process can go in both directions: reducing, or increasing the number of details.

Model mapping also allows to identify non-critical and critical parameters. Let us give only two examples. The constants or reactions 13,14 (formation of the complex) are not critical and one does not need to know them with precision. Actually, variations by a factor 100 in these constants do not change the dynamics.

The values that we use come from [51], who cites two other references [70,71] that seem to propose very different values. Our analysis shows that this is not important. On the contrary, we show that the constant *C*_0 _(total concentration of NF-*κ*B) is a critical parameter. Reference [72] proposes 60000 molecules in a volume (of a fibroblast cell) of 2000 *μm*^3^. This means *C*_0 _= 0.06 *μM*. Nevertheless, cell volume estimate is not really precise and errors can easily shift the model from a damped oscillatory to a sustained oscillatory dynamics. In this case, it is the comparison between model prediction and theoretical observation that can fix the value of the critical parameter.

The sequence of reduction steps described in this section is illustrated on Fig. 2S1–2S7 in Additional File 1. A series of models of decreasing complexity starting from ℳ(39, 65, 90) and upto ℳ(14, 25, 33) provided in SBML 2.1 [67] format and annotated by CellDesigner 3.5 software [68] are submitted to BioModels database  with the following ids: MODEL7743656488, MODEL7743631122, MODEL7743608569, MODEL7743576806, MODEL7743528808, MODEL7743444866.

## Conclusion

We have presented a methodology for reducing and comparing systems biology models. We show how to produce a hierarchy of coarse grained models that can be used for understanding functioning of the biological systems. We show how models in the hierarchy can be mapped one onto another, thus allowing to decrease or to increase the number of details that are needed for the description of the system. Our method identifies the set of critical parameters of the system. This can be particularly useful for robustness studies (when robustness is understood as stability against parameter variability [34]) or for practical multi-target approaches in pharmacology.

We did not approach aspects of multi-scale modeling that occur in multi-organ physiology, or spatial aspects. Relation with stochastic modelling has been only briefly discussed and will be presented in detail elsewhere (Crudu et al., in preparation). The reduction methods presented here can be applied to systems of biochemical reactions modeling cell physiology [73] and can be usefully applied to various problems in signalling, metabolism, genetic regulation.

A central idea in our treatment is the hypothesis that biological systems are hierarchial, involving many separated time scales. The reduction methods were adapted to exploit this situation (we look for dominant subsystems, which lead to tremendous simplification). The hierarchical nature of the systems is not sufficiently exploited by more traditional approaches. For instance, singular perturbation copes with two time scales and eliminates the fastest. In biology, we are often interested in a "middle" time-scale, corresponding to a particular process that we study. We have shown how to eliminate both faster and slower variables. Another specificity of systems biology is the quest for critical parameters. Our approach offers naturally a solution: critical parameters are detected in the reduction process. It also extends the theory of limiting step to complex networks [1]. Showing how to find critical parameters and dominant simplifications is a first step towards a dynamical systems approach to physiology. Indeed, complex networks fulfill various tasks in simple ways by activating a few degrees of freedom. Dominant subsystems gather dynamical variables that are activated and can change when the system needs to perform a given task. Changing task could be represented as zooming in and out (change the number of degrees of freedom), or jumping laterally (change the set of degrees of freedom) in the hierarchy of models. As pointed out by Denis Noble [74], physiology should not be understood from the bottom upwards. Our approach suggests that not only the subjective understanding, but also the objective functioning of biological systems can be based on middle-out (meaning variable level of detail) pictures.

As future work we will improve our algorithms in order to propose fully automated reduction tools. At present, the automated sections of our methods are the calculation of dominant subsystems of pseudo-monomolecular subsystems and the calculation of simple sub-mechanisms stoichiometries and rates. The detection of quasi-stationary and non-oscillating species is semi-automated. The solutions of quasi-stationarity and averaged stationarity equations are not yet fully automated (except for the pseudo-monomolecular case).

We also plan to consider other applications such as high dimensional switches [75].

Concerning our model comparison methods, we would like to study hierarchies of kinetic models coming from various organisms, for which the conserved and the specific parts are the result of evolution.

## Authors' contributions

OR proposed the methodology to reduce nonlinear models. AG developed the general theory of multiscale linear system, together with OR and AZ. AZ and OR designed and implemented the algorithms. AL designed the NF-*κ*B model. All authors drafted, read and approved the final manuscript.

## Supplementary Material

Additional file 1 Hierarchy of NF*κ*B models. This file shows the hierarchy of models using Systems Biology Graphical Notation (SBGN) and the results of a study of the truly non-linear reactions. Click here for file
