# Supplementary material for: Robust simplifications of multiscale biochemical networks
Source: BMC Syst Biol. 2008 Oct 14;2:86. doi: 10.1186/1752-0509-2-86 (PMC2654786; doi:10.1186/1752-0509-2-86)
Supplement: Additional file 1 — Hierarchy of NFκB models. This file shows the hierarchy of models using Systems Biology Graphical Notation (SBGN) and the results of a study of the truly non-linear reactions. [file 1752-0509-2-86-S1.ppt]

## Slide 1
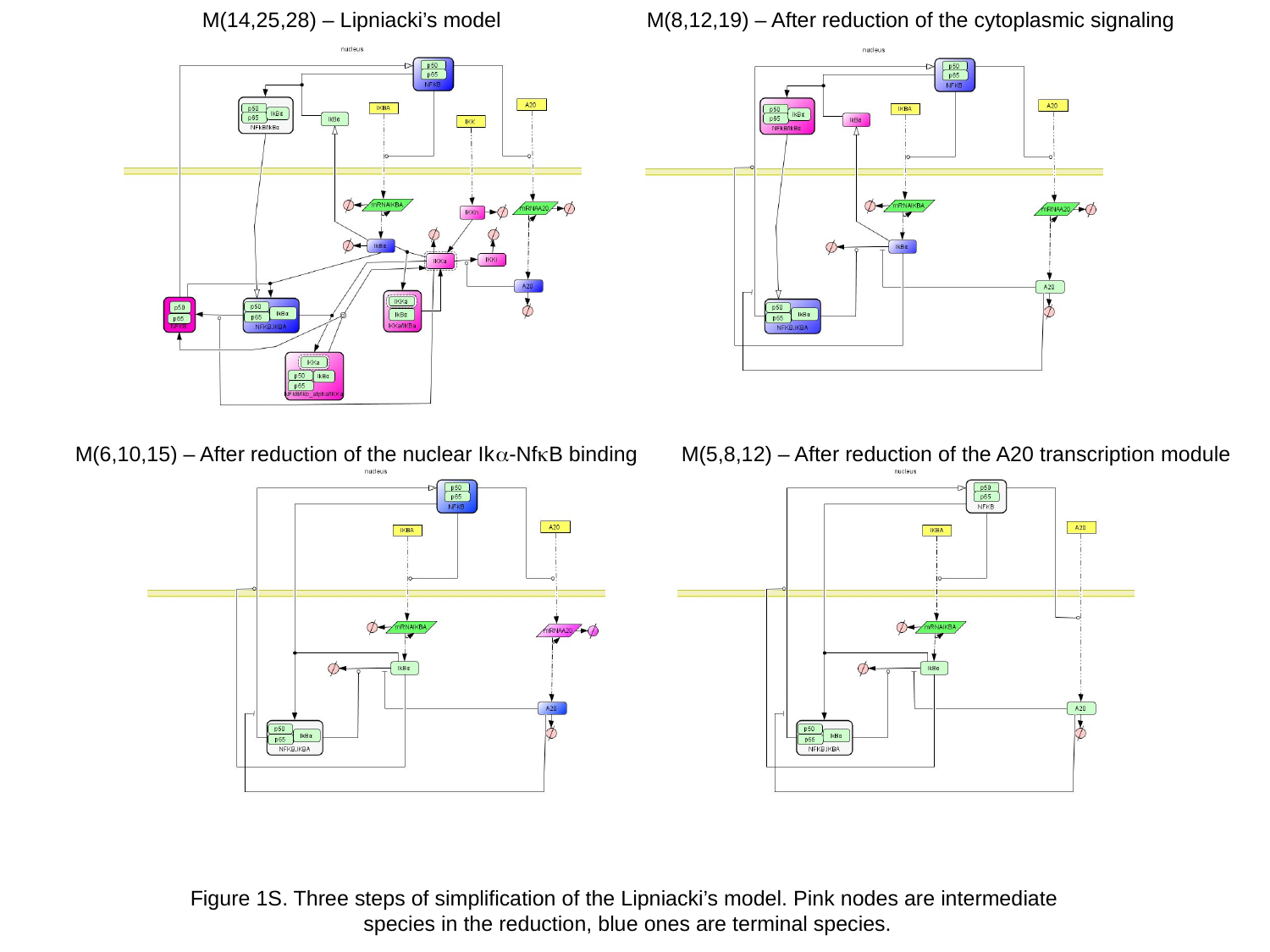

M(14,25,28) – Lipniacki’s model
M(8,12,19) – After reduction of the cytoplasmic signaling
M(6,10,15) – After reduction of the nuclear Ikα-NfκB binding
M(5,8,12) – After reduction of the A20 transcription module
Figure 1S. Three steps of simplification of the Lipniacki’s model. Pink nodes are intermediate
species in the reduction, blue ones are terminal species.

## Slide 2
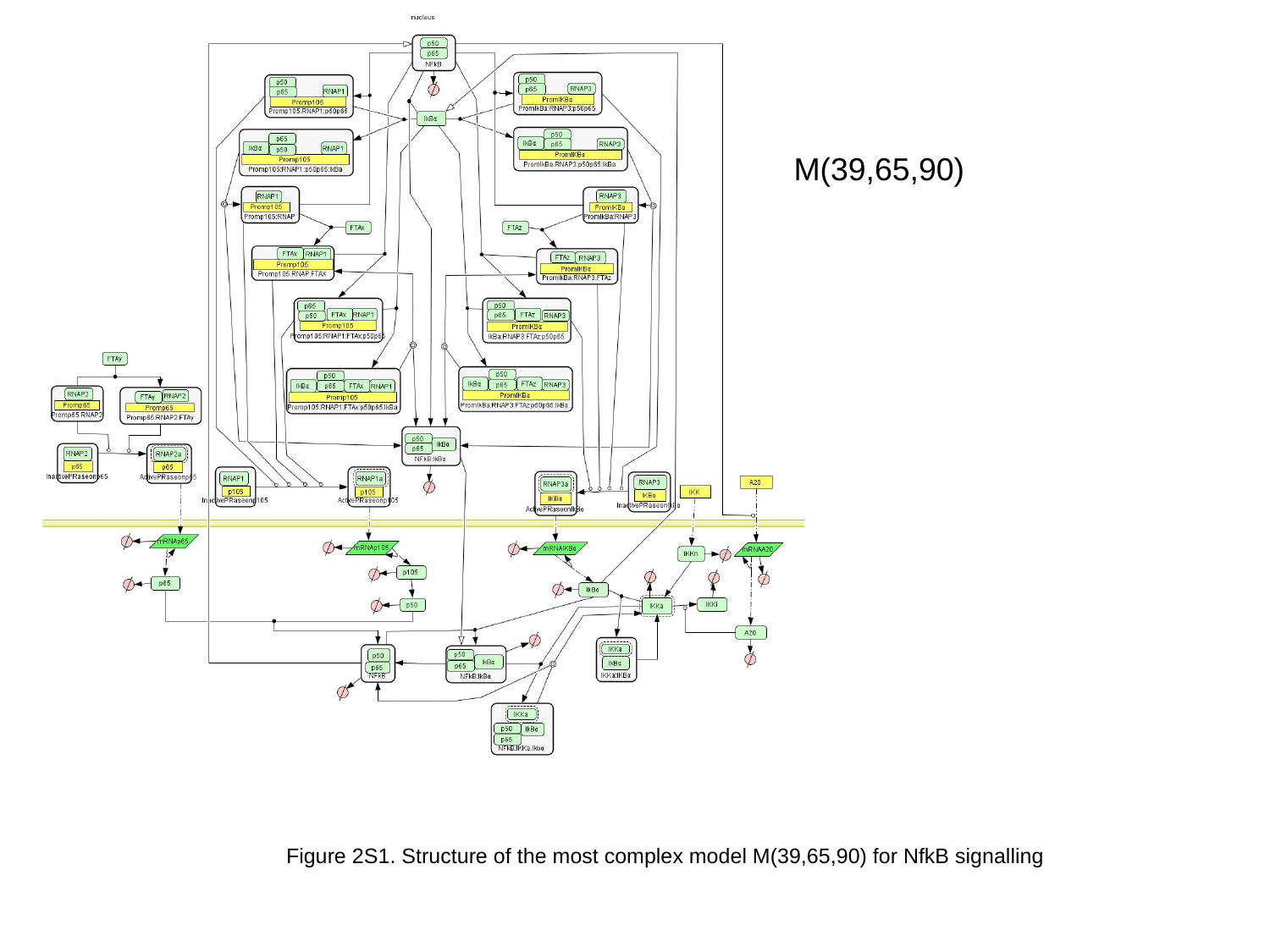

M(39,65,90)
Figure 2S1. Structure of the most complex model M(39,65,90) for NfkB signalling

## Slide 3
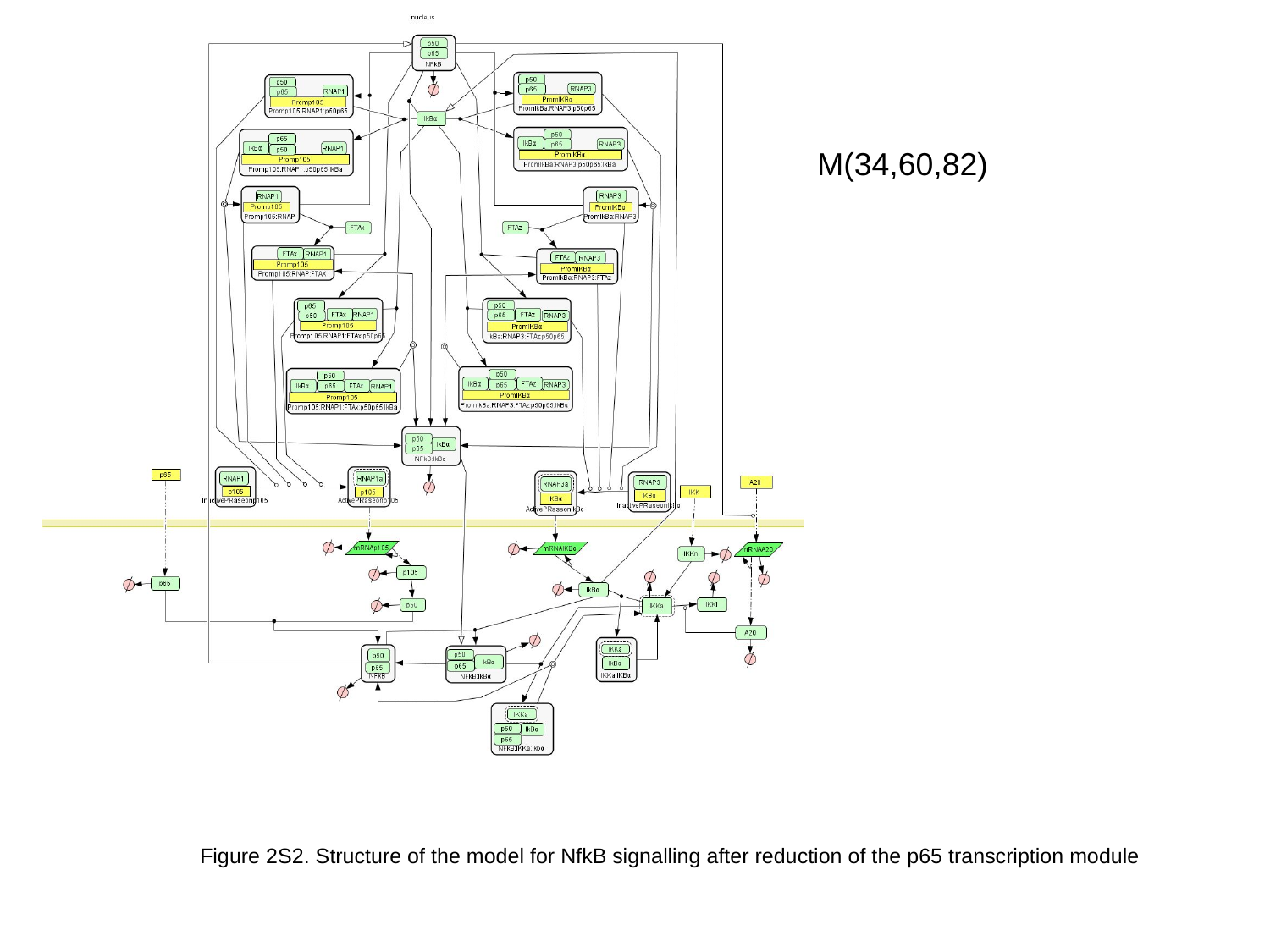

M(34,60,82)
Figure 2S2. Structure of the model for NfkB signalling after reduction of the p65 transcription module

## Slide 4
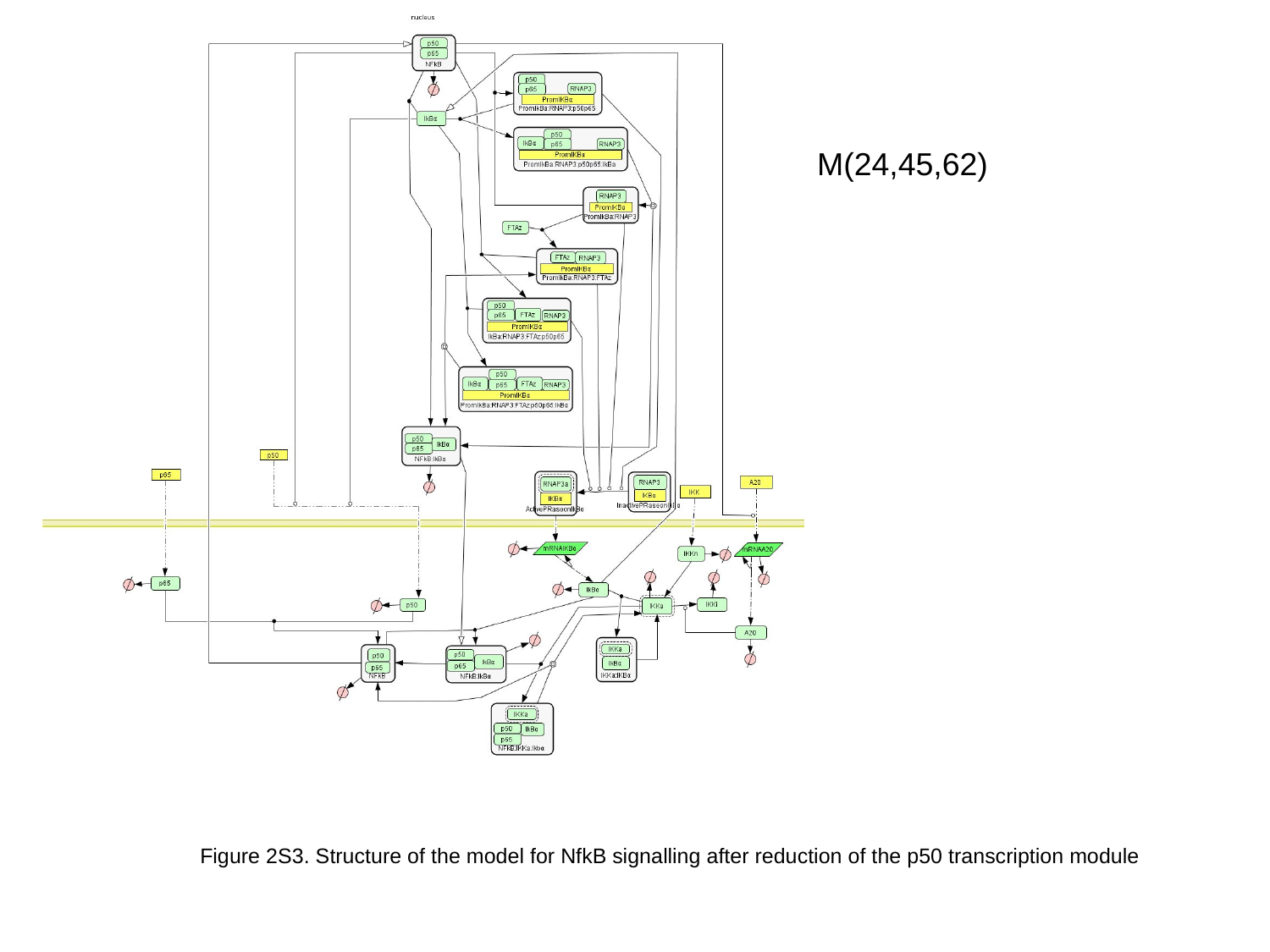

M(24,45,62)
Figure 2S3. Structure of the model for NfkB signalling after reduction of the p50 transcription module

## Slide 5
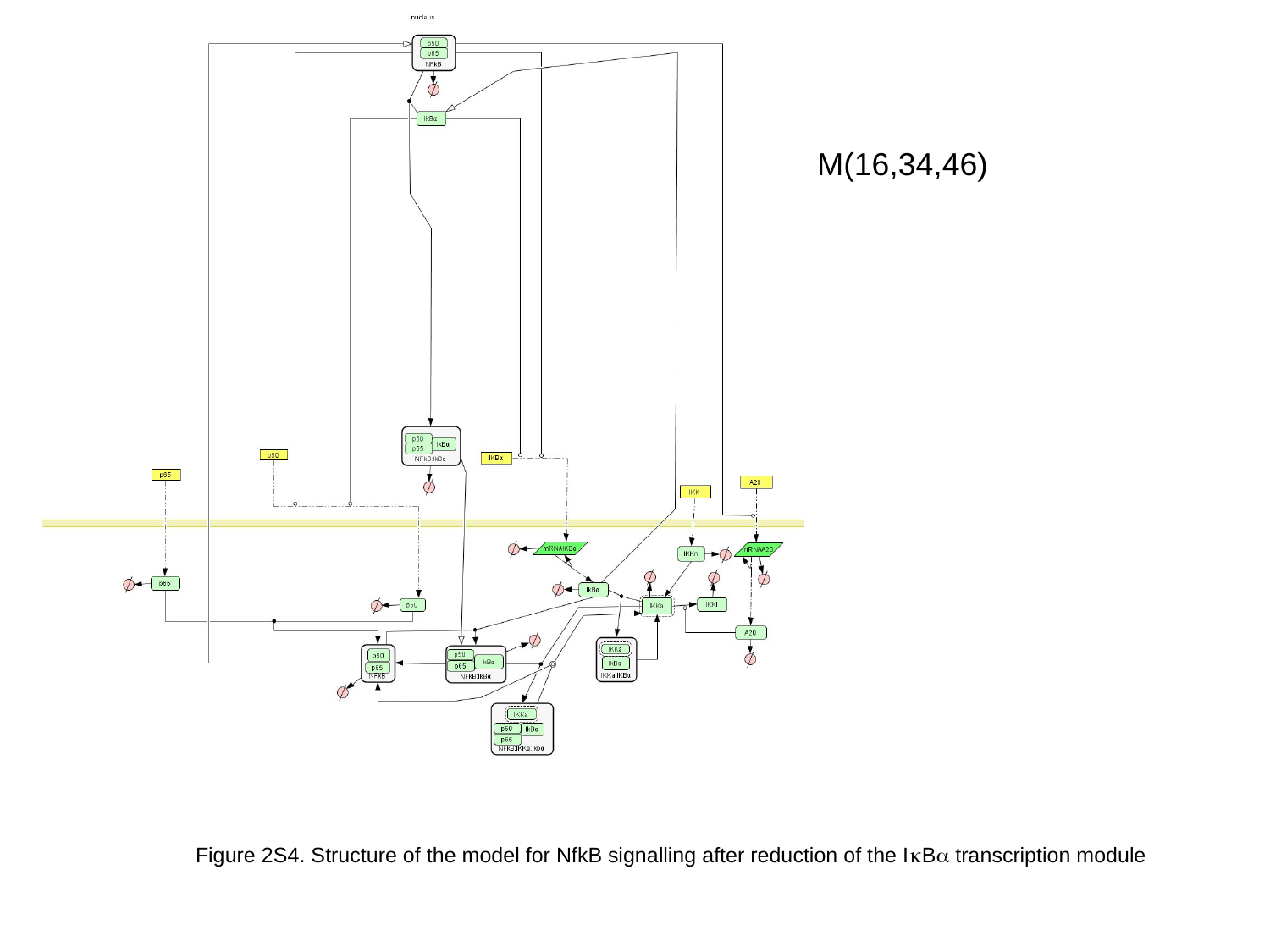

M(16,34,46)
Figure 2S4. Structure of the model for NfkB signalling after reduction of the IκBα transcription module

## Slide 6
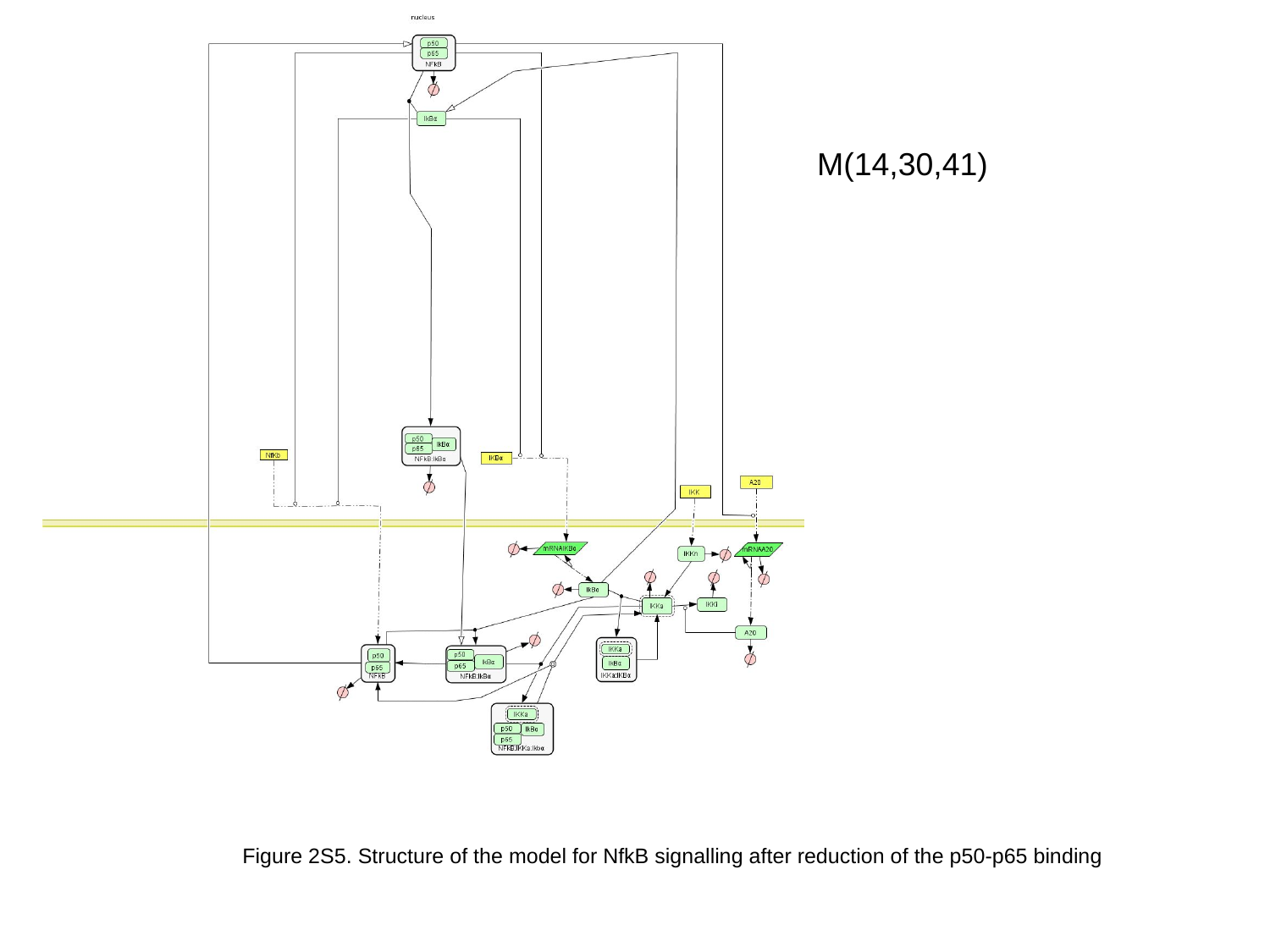

M(14,30,41)
Figure 2S5. Structure of the model for NfkB signalling after reduction of the p50-p65 binding

## Slide 7
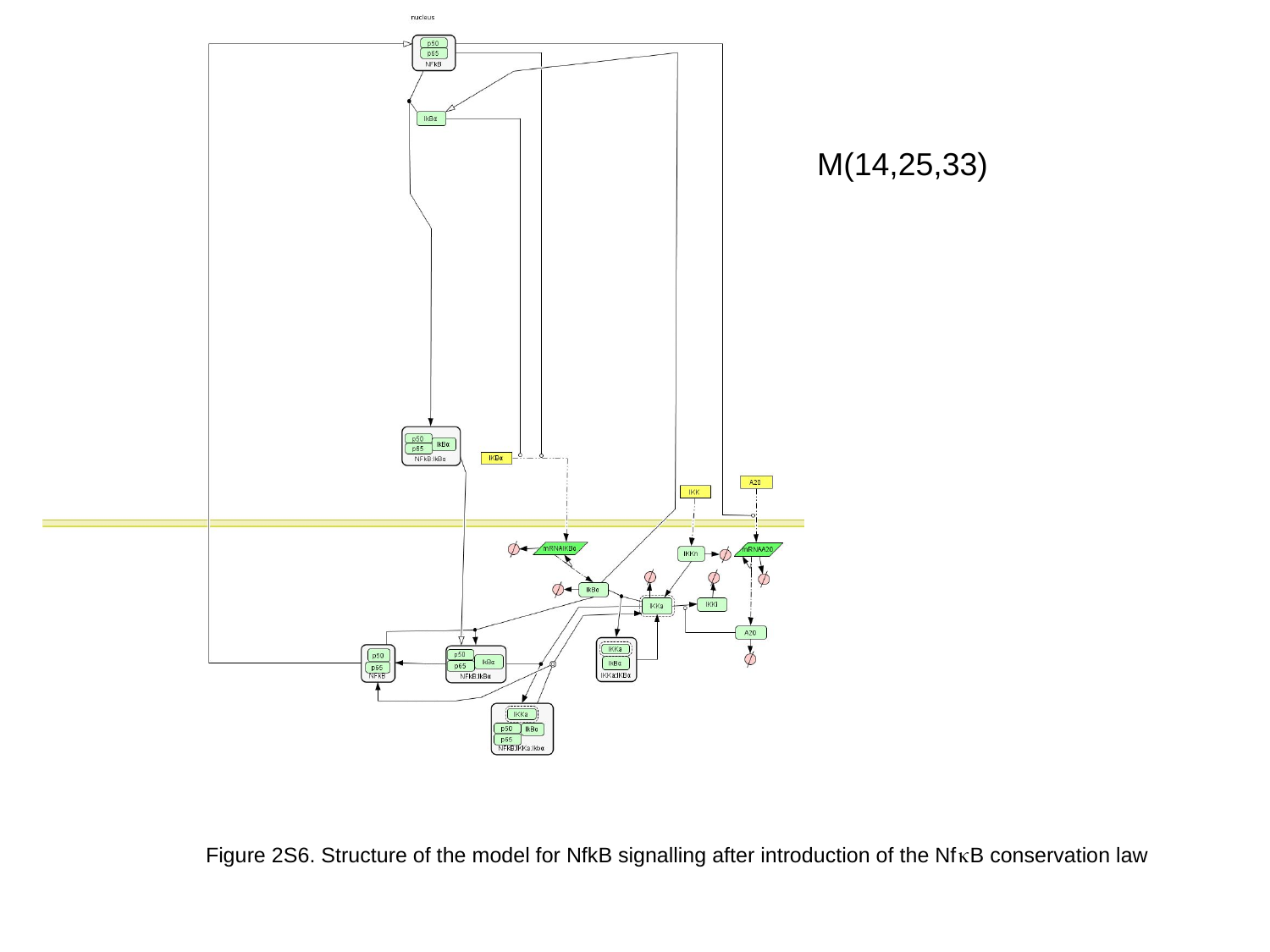

M(14,25,33)
Figure 2S6. Structure of the model for NfkB signalling after introduction of the NfκB conservation law

## Slide 8
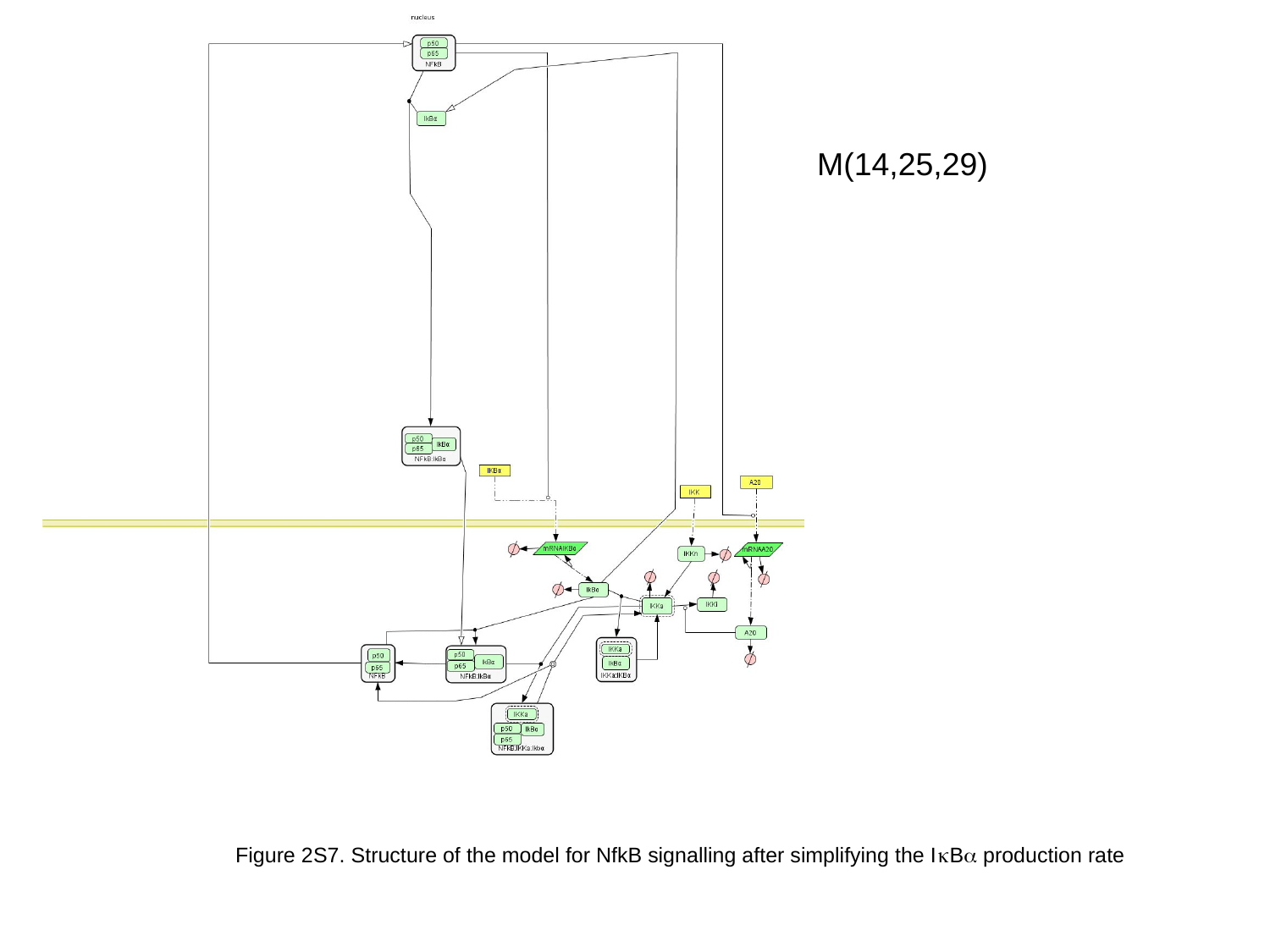

M(14,25,29)
Figure 2S7. Structure of the model for NfkB signalling after simplifying the IκBα production rate

## Slide 9
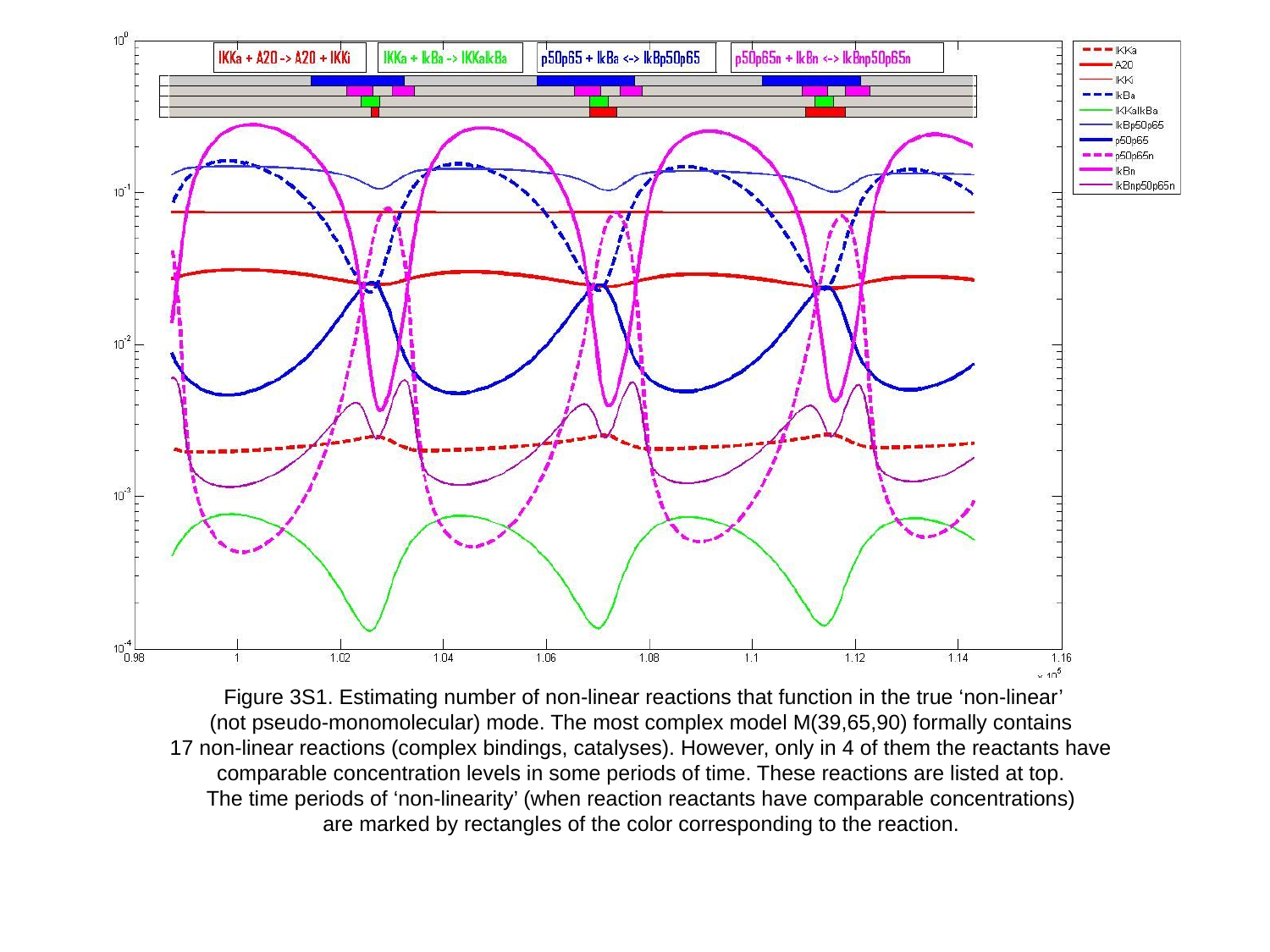

Figure 3S1. Estimating number of non-linear reactions that function in the true ‘non-linear’
(not pseudo-monomolecular) mode. The most complex model M(39,65,90) formally contains
17 non-linear reactions (complex bindings, catalyses). However, only in 4 of them the reactants have
comparable concentration levels in some periods of time. These reactions are listed at top.
The time periods of ‘non-linearity’ (when reaction reactants have comparable concentrations)
are marked by rectangles of the color corresponding to the reaction.

## Slide 10
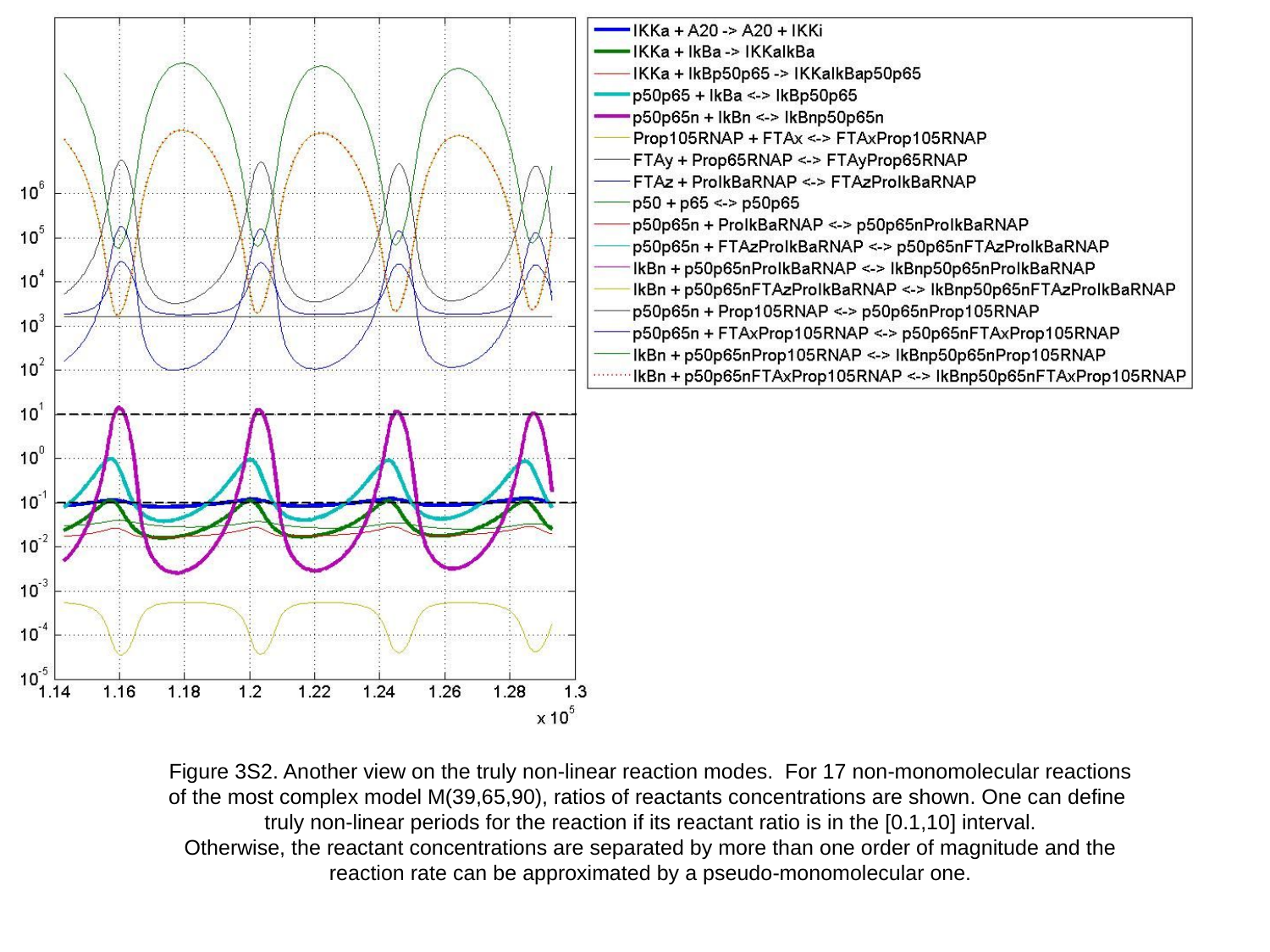

Figure 3S2. Another view on the truly non-linear reaction modes. For 17 non-monomolecular reactions
of the most complex model M(39,65,90), ratios of reactants concentrations are shown. One can define
truly non-linear periods for the reaction if its reactant ratio is in the [0.1,10] interval.
Otherwise, the reactant concentrations are separated by more than one order of magnitude and the
reaction rate can be approximated by a pseudo-monomolecular one.
